# Design, synthesis and evaluation of quinoline-*O*-carbamate derivatives as multifunctional agents for the treatment of Alzheimer’s disease

**DOI:** 10.1080/14756366.2023.2169682

**Published:** 2023-01-23

**Authors:** Hongsong Chen, Jing Mi, Sen Li, Zhengwei Liu, Jing Yang, Rui Chen, Yujie Wang, Yujuan Ban, Yi Zhou, Wu Dong, Zhipei Sang

**Affiliations:** a Inner Mongolia Key Laboratory of Toxicant Monitoring and Toxicology, College of Animal Science and Technology, Inner Mongolia Minzu University, Tongliao, Inner Mongolia, China; b College of Chemistry and Pharmaceutical Engineering, Nanyang Normal University, Nanyang, Henan, China; c Department of Orthopaedics Surgery, Nanyang Central Hospital, Nanyang, Henan, China; d State Key Laboratory of Functions and Applications of Medicinal Plants, Guizhou Provincial Engineering Technology Research Center for Chemical Drug R&D, Guizhou Medical University, Guiyang, Guizhou, China; e School of Pharmaceutical Sciences, Hainan University, Haikou, Hainan, China

**Keywords:** Alzheimer’s disease, quinoline-*O*-carbamate derivatives, multifunctional agent, zebrafish AD model

## Abstract

A series of novel quinoline-*O*-carbamate derivatives was rationally designed for treating Alzheimer’s disease (AD) by multi-target-directed ligands (MTDLs) strategy. The target compounds were synthesised and evaluated by AChE/BuChE inhibition and anti-inflammatory property. The in vitro activities showed that compound **3f** was a reversible dual *ee*AChE/eqBuChE inhibitor with IC_50_ values of 1.3 µM and 0.81 µM, respectively. Moreover, compound **3f** displayed good anti-inflammatory property by decreasing the production of IL-6, IL-1β and NO. In addition, compound **3f** presented significant neuroprotective effect on A*β*_25-35_-induced PC12 cell injury. Furthermore, compound **3f** presented good stabilities in artificial gastrointestinal fluids, liver microsomes *in vitro* and plasma. Furthermore, compound **3f** could improve AlCl_3_-induced zebrafish AD model by increasing the level of ACh. Therefore, compound **3f** was a promising multifunctional agent for the treatment of AD.

## Introduction

Alzheimer’s disease (AD) is the main cause of dementia and is quickly becoming one of the most expensive, lethal, and burdening disease of this century^1^. At present, more than 50 million people worldwide are affected and the figure of the AD patients will triple by 2050^2^.

The pathologic changes of AD are convoluted and multilayered, these abnormal changes include the low levels of acetylcholine (ACh), loss of synapses, neuroinflammation, aberrant accumulation of amyloid-*β* (A*β*) oligomer, neurofibrillary tangles within neurons and elevated oxidative stress^3^. Presently, the primary therapeutic options for AD treatment are acetylcholinesterase inhibitors (AChEIs), such as donepezil, rivastigmine, galantamine, as well as the *N*-methyl-*D*-aspartate (NMDA) receptor antagonist, memantine, which can improve memory and cognitive function to a certain extent but cannot halt or reverse the progression of this disease^4^^,^^5^. Due to the complexity of AD and the involvement of multi-systems in the process of AD, the multi-target-directed ligands (MTDLs) approach, that is one molecule hits two or more AD-relevant complementary targets and produces synergistic effect on the disease network, has been developed in both symptomatic and disease-modifying efficiencies^5^^,^^6^.

The “cholinergic hypothesis” states that loss of cholinergic function is the important biomarkers of AD. It has been verified that the high levels of AChE facilitate the hydrolysation of ACh lead to memory deficit in the early stages of AD^7^^,^^8^. Moreover, AChE produces promotion in Aβ deposition in the form of senile plaques in the brain of afflicted individuals^9^. BuChE is an enzyme closely related to AChE and serves as a co-regulator of cholinergic neurotransmission by hydrolysing ACh. It is fact that the AChE level in the brain declines progressively as the progress of AD goes, while BChE level soars to 165% of the normal level in the late stages of AD, this implies BChE takes over the hydrolysis of ACh in late AD^10^. An increased BuChE activity also plays an important role in A*β*-aggregation during the early stages of senile plaque formation^11^. Thus, dual AChE/BuChE inhibition have been documented as effective management for the treatment of AD by an increase in the availability of ACh in the brain regions and decrease in the A*β* deposition, such as rivastigmine.

Investigations suggest that inflammation clearly occurs in pathologically vulnerable regions of the AD brain, which related to microglia, astrocytes and inflammatory factors. In the AD brain, damaged neurons and neurites and highly insoluble A*β* oligomer and neurofibrillary tangles provide obvious stimuli for inflammation, such as tumour necrosis factor (TNF-α), IL-6, IL-1*β* and NO. AD inflammation significantly contributed to AD pathogenesis^12–14^. Therefore, to develop anti-inflammatory agent could provide an effective approach to slow the progression or delay the onset of AD.

**Figure 1.**
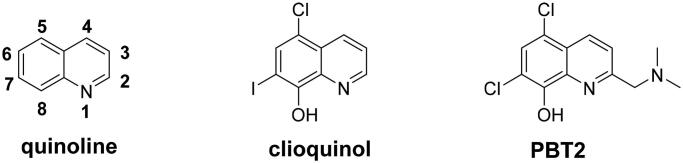
The structure of quinoline, clioquinol and PBT2.

**Figure 2.**
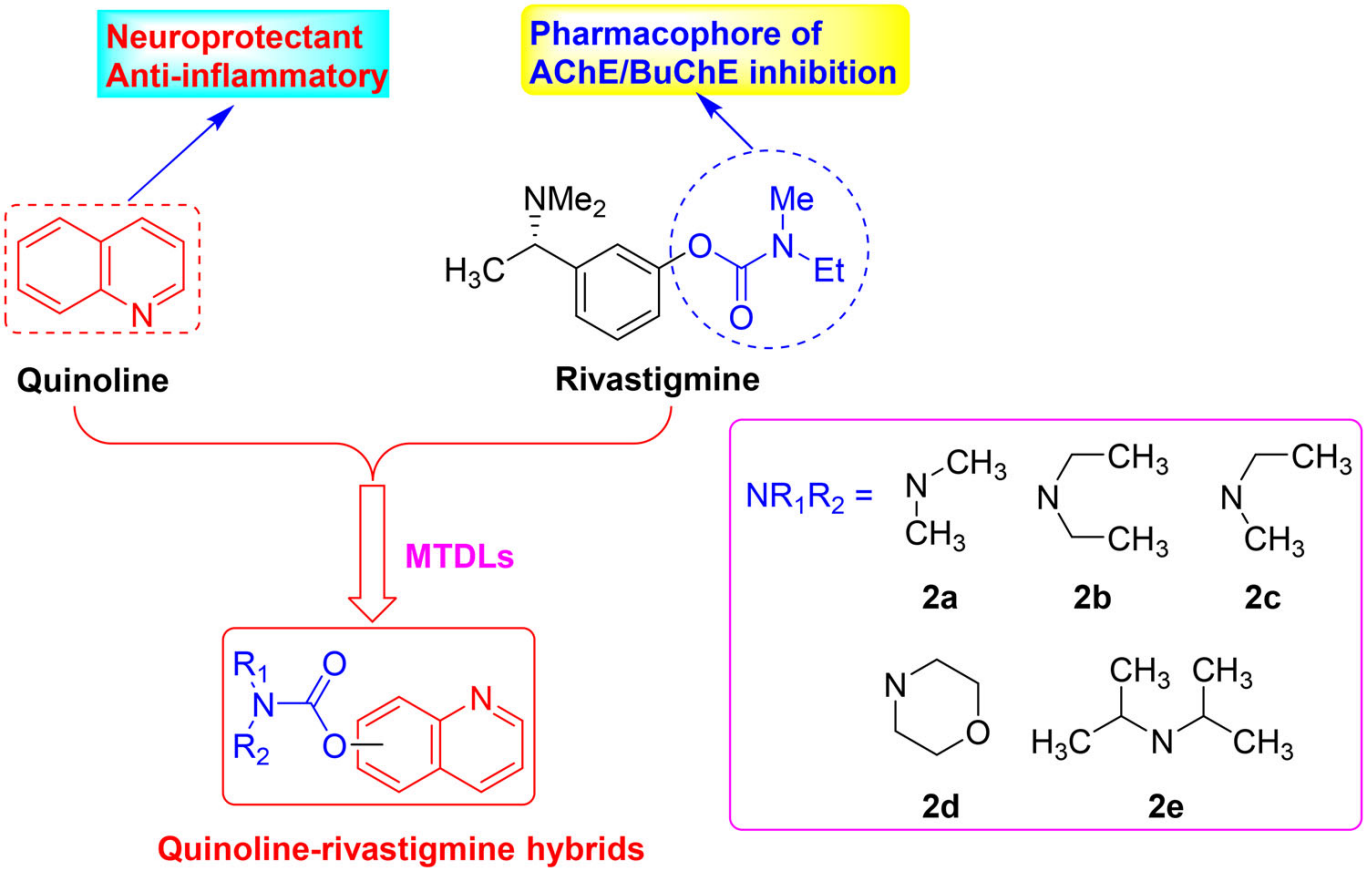
The design strategy of quinoline-rivastigmine hybrids.

Quinoline core (Figure 1), a bicyclic structure in which a pyridinic ring is fused to a benzene moiety, occurs in several natural compounds (*Cinchona* alkaloids) and in drugs, with a broad range of biological activities including antimicrobial, antifungal, antitumor, antiviral, anticholesterol and anti-Alzheimer’s disease^15^^,^^16^. The 8-hydroxy quinoline derivatives clioquinol and PBT2 (Figure 1), promising multifunctional agents, have been investigated for their neuroprotective effect in AD and have entered clinical trials for AD^17^. Other quinoline derivatives also show AChE/BuChE inhibitory potency, antioxidant activity, metal chelators, anti-inflammatory potency and inhibition effects on A*β* aggregation^18–20^. Moreover, rivastigmine is a dual AChE/BuChE inhibitor approved by FDA for the treatment of mild to moderate AD^21^. The carbamate moiety is the pharmacophore, and many carbamate derivatives have been developed as multifunctional agents anti-AD by our group and other groups^22–25^. Herein, we plan to introduce carbamate moiety into the promising quinoline skeleton to obtain novel quinoline-rivastigmine hybrids by MTDLs (Figure 2). The synthesised target compounds are evaluated through *in vitro* and in vivo and hope to get a promising multifunctional agent to treat AD.

## Results and discussion

### Chemistry

**Scheme 1.**
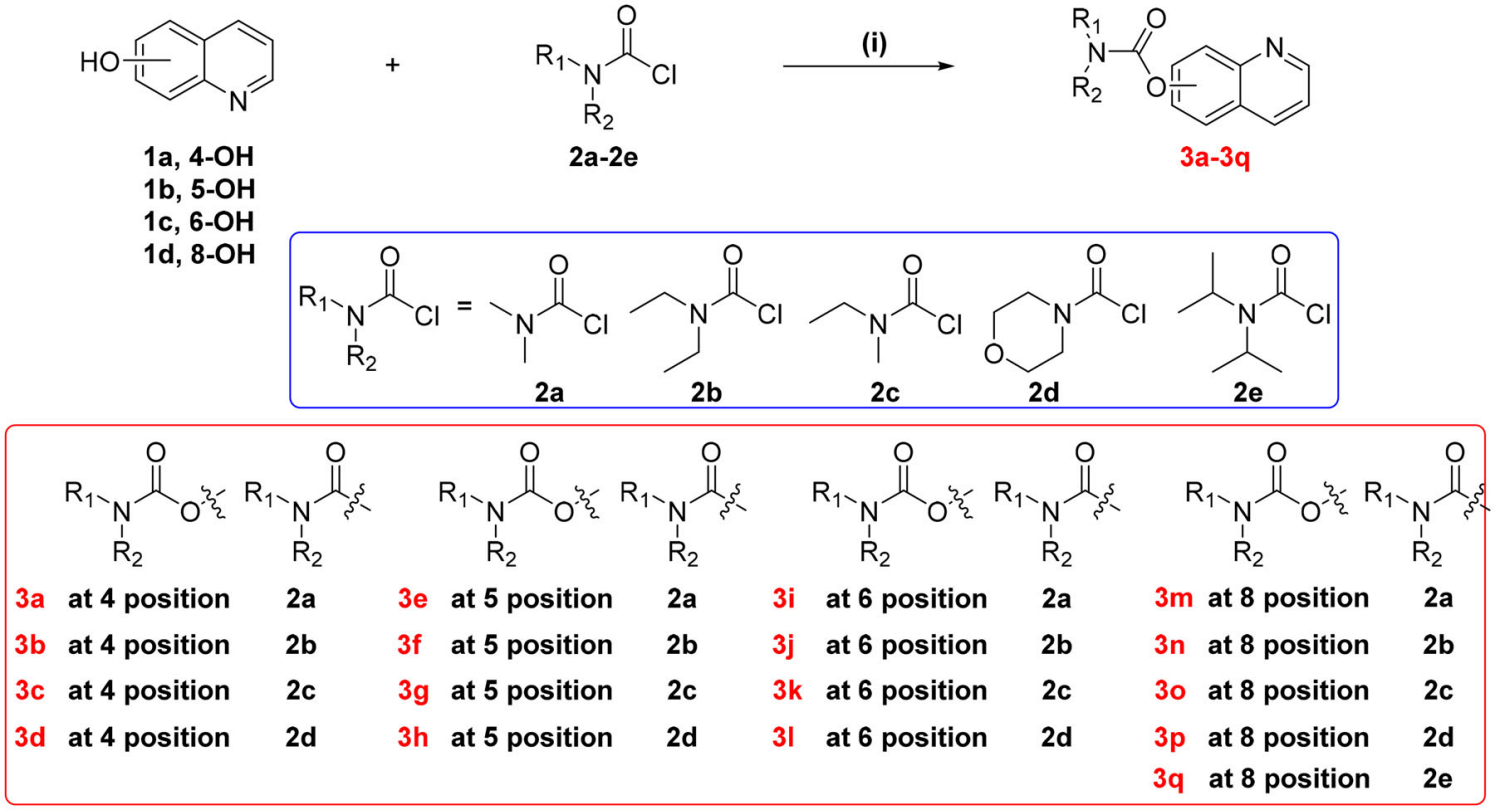
Synthesis of target compounds **3a-3q**. Reagents and conditions: (i) 1.3 equivalent *N,N*-disubstituted carbamoyl chlorides (**2a-2e**), 1.5 equivalent K_2_CO_3_, CH_3_CN, 65 °C, for 6**–**10 h.

The synthetic route of target derivatives **3a-3q** were summarised in Scheme 1. Briefly, the starting material **1a–1d** (4-hydroxyquinoline **1a**, 5-hydroxyquinoline **1b**, 6-hydroxyquinoline **1c**, and 8-hydroxyquinoline **1d**) were treated with excessive amounts of dimethylcarbamoyl chloride (**2a**), diethylcarbamyl chloride (**2b**), *N*-ethyl-*N*-methylcarbamoyl chloride (**2c**), 4-morpholinecarbonyl chloride (**2d**), and diisopropylcarbamoyl chloride (**2e**), respectively, in the presence of K_2_CO_3_ in CH_3_CN at 60–65 °C to obtain the target compounds **3a–3q**. All the target compounds were purified by chromatography, and the analytical and spectroscopic data confirmed their structures, as detailed in the Supporting Information.

### Biological activity

#### Inhibition effects on AChE and BuChE

The inhibitory activities of the synthesised compounds against *ee*AChE (from *electric eel*) and eqBuChE (from *equine serum*) were tested by the modified Ellman’s method^26^^,^^27^. Rivastigmine and donepezil were used as positive compounds. As displayed in Table 1, both the carbamate fragments and the position of carbamate fragment significantly influenced *ee*AChE/eqBuChE inhibitory potency. Overall speaking, compounds **3a–3d** with carbamate fragments at 4-position of quinoline showed significantly selective eqBuChE inhibitory potency, especially, compound **3d** showed the best eqBuChE inhibitory potency with IC_50_ value of 0.87 µM; When the carbamate fragments at 5-position of quinoline, the compounds **3e–3h** were potent dual AChE/BuChE inhibitors, particularly, compound **3f** showed excellent dual AChE/BuChE inhibitory activity with IC_50_ values of 1.3 µM and 0.81 µM, respectively; When the carbamate fragments at 6-position of quinoline, compounds **3i–3l** showed moderate selective *ee*AChE inhibitory potency, particularly, compound **3k** showed moderate *ee*AChE inhibitory activity with IC_50_ value of 10.3 µM; When the carbamate fragments at 8-position of quinoline, compounds **3m–3q** were potent selective *ee*AChE inhibitors, particularly, compound **3m** showed good *ee*AChE inhibitory activity with IC_50_ value of 6.5 µM.

**Table 1.** AChE/BuChE inhibitory activities by target compounds **3a–3q**, rivastigmine, and donepezil.

Compd	IC_50_ ± SD^*a*^ (μM)		SI^*d*^	IC_50_ ± SD^*a*^ (μM)		SI^*d*^
	*ee*AChE^*b*^	eqBuChE^*c*^		hAChE^*e*^	hBuChE^*f*^	
**3a**	10.8 ± 0.33	2.8 ± 0.09	3.8	NT^*h*^	NT^*h*^	–
**3b**	12.7 ± 0.13	1.1 ± 0.13	11.5	30.2 ± 0.61	5.4 ± 0.06	5.6
**3c**	13.2 ± 0.51	1.9 ± 0.07	6.9	NT^*h*^	NT^*h*^	–
**3d**	15.7 ± 0.27	0.87 ± 0.05	18.0	26.9 ± 0.74	9.5 ± 0.07	2.8
**3e**	4.5 ± 0.04	0.94 ± 0.07	4.8	10.4 ± 0.21	3.7 ± 0.05	2.8
**3f**	1.3 ± 0.02	0.81 ± 0.06	1.6	5.6 ± 0.07	2.3 ± 0.03	2.4
**3g**	3.2 ± 0.05	2.2 ± 0.08	1.4	NT^*h*^	NT^*h*^	–
**3h**	12.9 ± 0.07	1.8 ± 0.05	7.2	NT^*h*^	NT^*h*^	–
**3i**	14.1 ± 0.43	13.8 ± 0.61%^*g*^	–	NT^*h*^	NT^*h*^	–
**3j**	12.2 ± 0.19	30.7 ± 1.26^*g*^	–	NT^*h*^	NT^*h*^	–
**3k**	10.3 ± 0.58	5.8 ± 0.03%^*g*^	–	NT^*h*^	NT^*h*^	–
**3l**	17.6 ± 0.61	13.8 ± 0.61%^*g*^	–	NT^*h*^	NT^*h*^	–
**3m**	6.5 ± 0.05	7.3 ± 0.05%^*g*^	–	NT^*h*^	NT^*h*^	–
**3n**	9.7 ± 0.07	2.4 ± 0.01%^*g*^	–	NT^*h*^	NT^*h*^	–
**3o**	8.2 ± 0.06	4.1 ± 0.02%^*g*^	–	NT^*h*^	NT^*h*^	–
**3p**	12.6 ± 0.76	10.8 ± 0.07%^*g*^	–	NT^*h*^	NT^*h*^	–
**3q**	14.8 ± 0.68	1.7 ± 0.01%^*g*^	–	NT^*h*^	NT^*h*^	–
**Rivastigmine**	10.2 ± 0.43	4.9 ± 0.33	2.1	7.9 ± 0.21	2.8 ± 0.52	2.8
**Donepezil**	0.017 ± 0.003	5.6 ± 0.31	0.003	0.018 ± 0.0004	4.3 ± 0.06	0.004

*^a^*Values are expressed as the mean ± standard deviation of the mean by three independent experiments in triplicate.

*^b^ee*AChE was from electric eel AChE.

*^c^eq*BChE from *equine serum*.

*^d^*SI = Selectivity Index = IC_50_ (AChE)/IC_50_ (BuChE).

*^e^*From human erythrocytes.

*^f^*From human serum.

*^g^*eqBuChE inhibition rate at 25 μM of compounds.

*^h^*NT = not tested.

As for the inhibitory effect of carbamate fragment, when the carbamate fragment at the 4-position of quinoline, compound **3a-3d** were selective eqBuChE inhibitors, compound **3a** with dimethylcarbamoyl fragment showed good eqBuChE inhibitory potency with IC_50_ value of 2.83 µM. When the dimethylcarbamoyl of **3a** was replaced with diethylcarbamoyl to get compound **3b**, the eqBuChE inhibitory potency increased to 1.06 µM. When the dimethylcarbamoyl of **3a** was replaced with *N*-ethyl-*N*-methylcarbamoyl to get compound **3c**, the eqBuChE inhibitory potency increased to 1.92 µM. When the dimethylcarbamoyl of **3a** was replaced with 4-morpholinecarbonyl to obtain compound **3d**, the eqBuChE inhibitory potency increased to 0.87 µM. When the carbamate fragment at the 5-position of quinoline, compound **3e** with dimethylcarbamoyl fragment, compound **3f** with diethylcarbamoyl fragment, and compound **3g** with *N*-ethyl-*N*-methylcarbamoyl fragment were good dual *ee*AChE/eqBuChE inhibitors with IC_50_ values of 4.5 µM/0.94 µM, 1.3 µM/0.81 µM and 3.2 µM/2.23 µM, respectively, while compound **3h** with 4-morpholinecarbonyl showed moderate *ee*AChE inhibitory activity (IC_50_ = 12.9 µM) and good eqBuChE inhibitory potency (IC_50_ = 1.76 µM). When the carbamate fragment at the 6-position of quinoline, compounds **3i-3l** showed moderate *ee*AChE inhibitory activities (ranging from 10.3 µM to 17.6 µM) and weak eqBuChE inhibitory activities. When the carbamate fragment at the 8-position of quinoline, compounds **3m-3q** showed moderate to good *ee*AChE inhibitory activities (ranging from 6.5 µM to 14.8 µM) and weak eqBuChE inhibitory activities. Compound **3m** with dimethylcarbamoyl fragment showed good eeAChE inhibitory potency (IC_50_ = 6.5 µM). When the dimethylcarbamoyl of **3m** was replaced with diethylcarbamoyl and *N*-ethyl-*N*-methylcarbamoyl, respectively, to get compounds **3n** and **3o**, respectively, the *ee*AChE presented slightly decrease with IC_50_ values of 9.7 µM and 8.2 µM, respectively. While, when the dimethylcarbamoyl of **3m** was replaced with 4-morpholinecarbonyl and diisopropylcarbamoyl, respectively, to get compounds **3p** and **3q**, respectively, the *ee*AChE sharply decreased to 12.6 µM and 14.8 µM, respectively.

Furthermore, the representative compounds (**3b, 3d, 3e** and **3f**) with good *ee*AChE or eqBuChE inhibitory activity were re-evaluated using hAChE and hBChE. As shown in Table 1, overall speaking, the selective eqBuChE inhibitors **3b** and **3d** showed good *hu*BuChE inhibitory potency with IC_50_ values of 5.39 µM and 9.46 µM, respectively and weak *ee*AChE inhibitory potency. The dual *ee*AChE/eqBuChE inhibitors **3e** and **3f** showed good *hu*BuChE inhibitory potency and hAChE inhibitory potency, particularly, compound **3f** presented good dual hAChE/hBuChE inhibitory potency with IC_50_ values of 5.6 µM and 2.34 µM, respectively, deserving for further investigation.

#### Anti-inflammatory property

According to the ChEs inhibitory activity, compounds **3a**, **3c**, **3e**, **3f**, **3k**, **3m** and **3n** were selected to tested the anti-inflammatory potency by measuring the production of inflammatory mediators IL-6, IL-1β and NO in LPS-induced BV-2 cells^28^^,^^29^.

**Figure 3.**
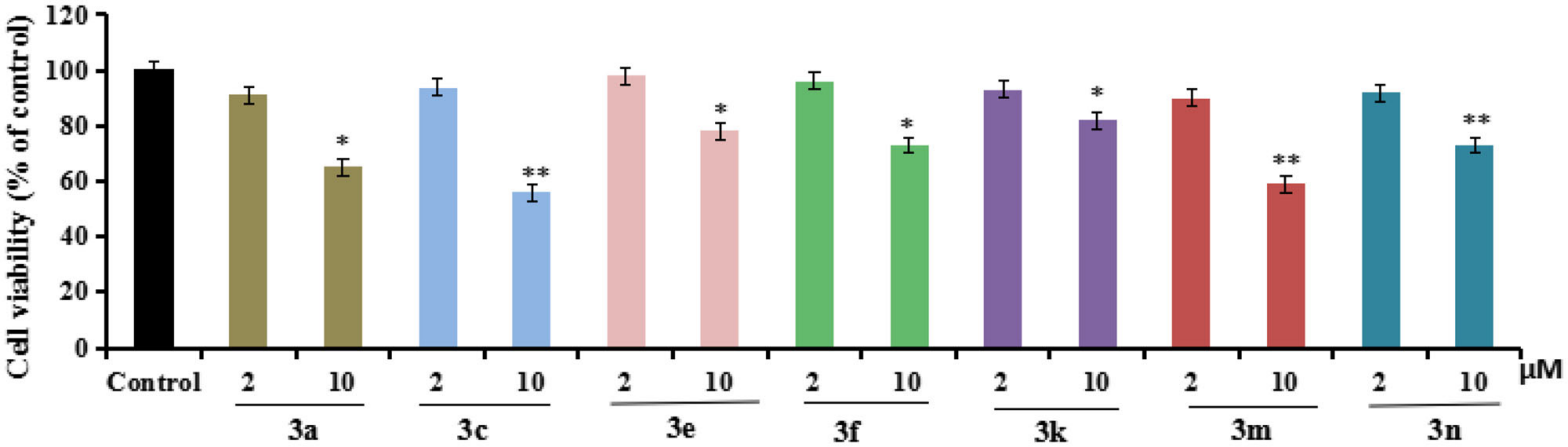
The cell viability of compounds **3a**, **3c**, **3e**, **3f**, **3k**, **3m,** and **3n** on the BV-2 cells, which was determined using MTT assay. The data are expressed as the mean ± SD by three independent experiments.

**Figure 4.**
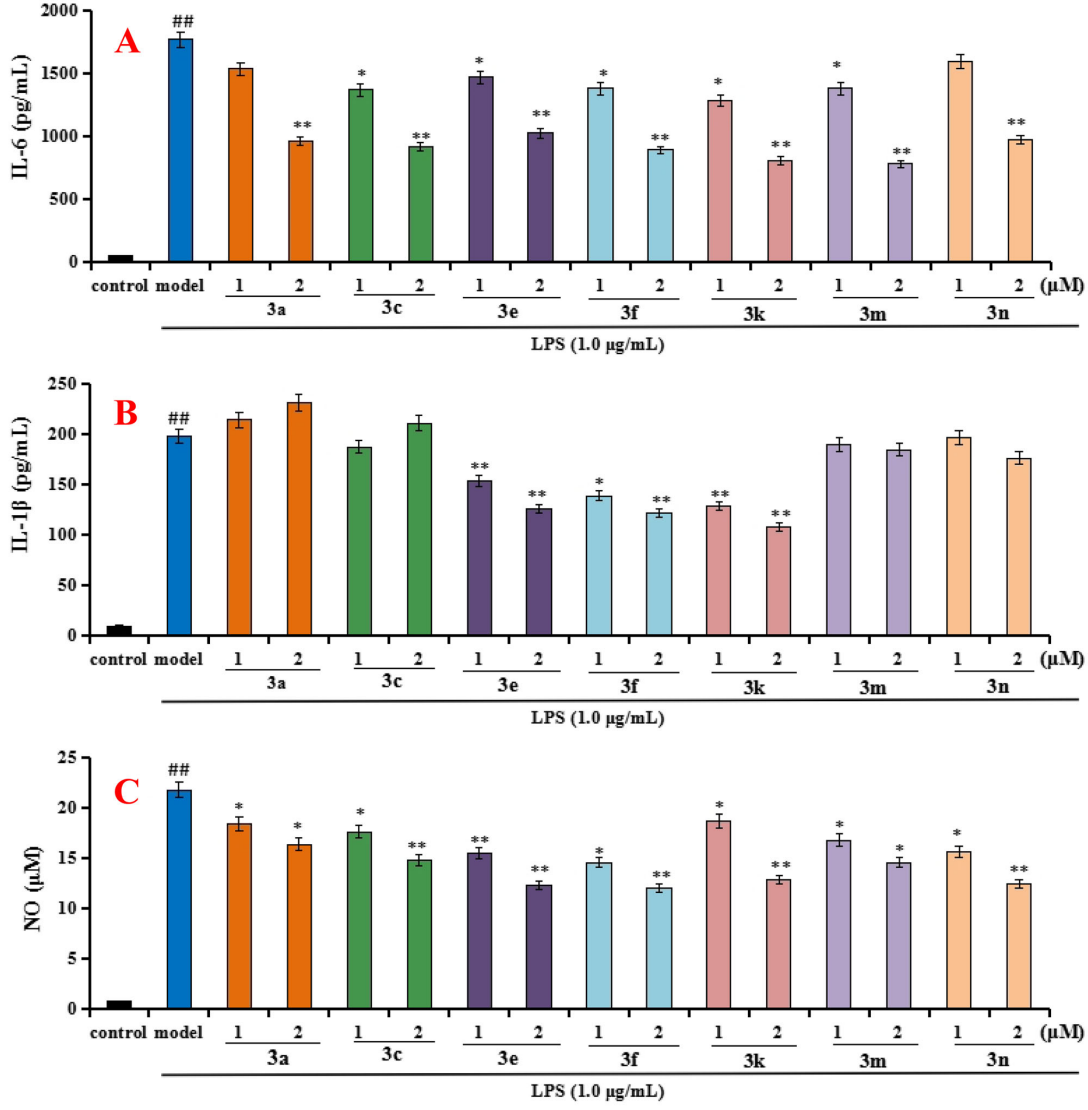
Effects of compounds **3a**, **3c**, **3e**, **3f**, **3k**, **3m,** and **3n** on the production of IL-6, IL-1β and NO in LPS-stimulated BV-2 cells. (A) Effects of compounds **3a**, **3c**, **3e**, **3f**, **3k**, **3m,** and **3n** on the production of IL-6; (B) Effects of compounds **3a**, **3c**, **3e**, **3f**, **3k**, **3m,** and **3n** on the production of IL-1β; (C) Effects of compounds **3a**, **3c**, **3e**, **3f**, **3k**, **3m,** and **3n** on NO release. Data were expressed as mean ± SD through three independent experiments. ##*p* < 0.01 *vs* control; **p < 0.01, *p < 0.05 *vs* LPS-induced model group.

1. Cytotoxicity of compounds **3a**, **3c**, **3e**, **3f**, **3k**, **3m** and **3n** on BV-2 cells. Firstly, the cytotoxicity of compounds **3a**, **3c**, **3e**, **3f**, **3k**, **3m** and **3n** were tested using CCK8 assay. As displayed in Figure 3, the cell viability did not show obvious change after adding compounds **3a**, **3c**, **3e**, **3f**, **3k**, **3m,** and **3n** at 2 µM.
2. Evaluation the production of IL-6, IL-1β and NO in LPS-stimulated BV-2 cells. As shown in Figure 4, when BV-2 cells were exposed to 1 µg/mL LPS for 24h, the levels of IL-6, IL-1β and NO significantly increased (*p* < 0.01), suggesting that the inflammatory model of BV-2 cells was successfully created and worked well. As displayed in Figure 4(A), compounds **3a**, **3c**, **3e**, **3f**, **3k**, **3m** and **3n** (at 1 µM and 2µM, respectively) could significantly decreased the levels of IL-6 compared with model group. As displayed in Figure 4(B), compounds **3e**, **3f** and **3k** could significantly decreased the levels of IL-1β compared with model group, while compounds **3a**, **3c**, **3m** and **3n** did not obviously decrease the level of IL-1β compared with model group. As displayed in Figure 4(C), compounds **3a**, **3c**, **3e**, **3f**, **3k**, **3m** and **3n** could significantly decreased the release volume of NO. The results exhibited that compounds **3e**, **3f**, **3k** showed significant anti-inflammatory activity by decreasing the production of inflammatory mediators IL-6, IL-1β and NO.

#### hAChE/hBuChE reversibility of inhibition by 3f

**Figure 5.**
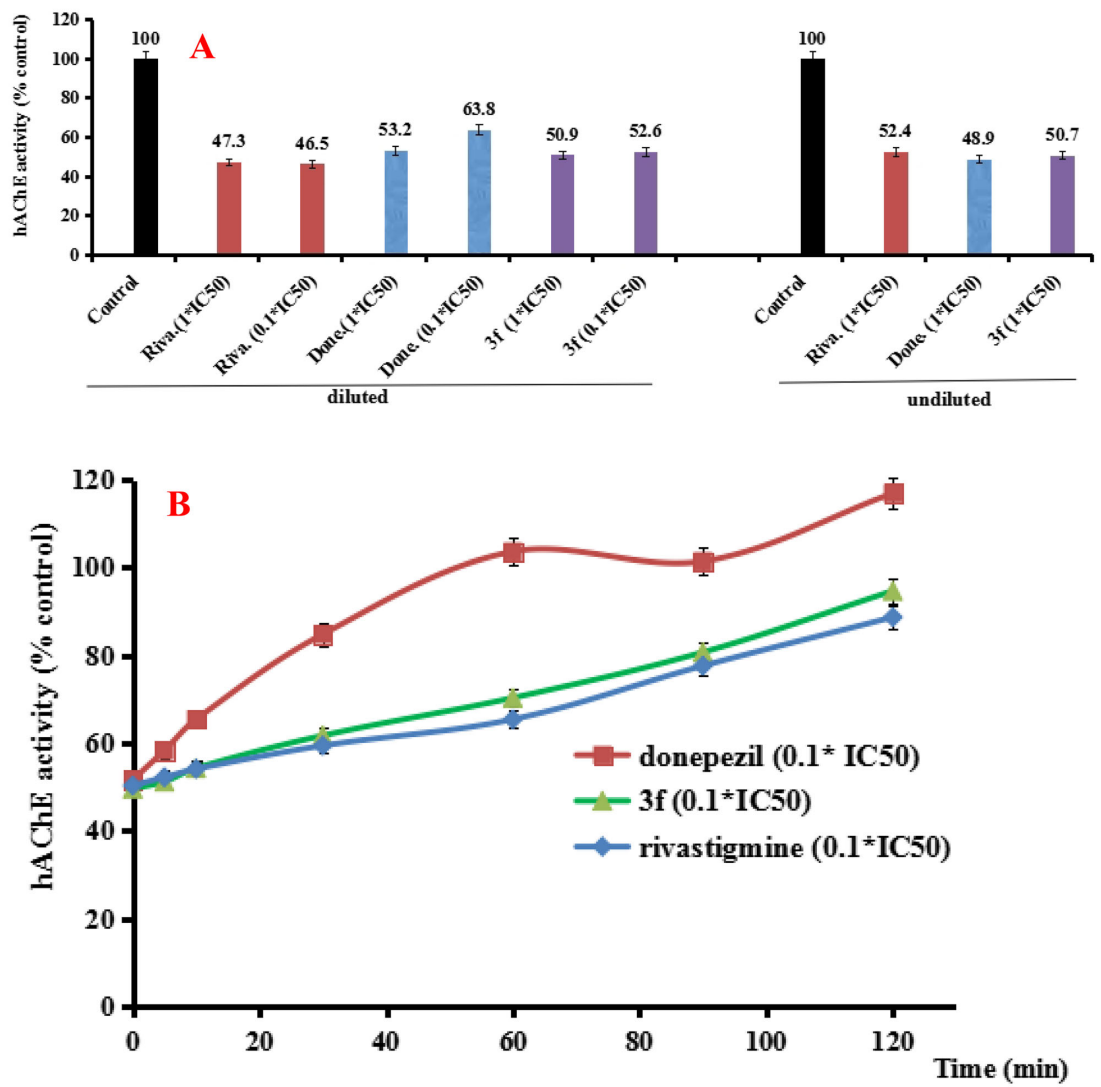
(A) hAChE recovery after preincubation of compound **3f** diluted to 1× or 0.1 × IC_50_, compared to donepezil (done.) diluted, rivastigmine (riva.) diluted and undiluted inhibition. (B) hAChE recovery of donepezil, **3f** diluted to 0.1 × IC_50_, were monitored with time at room temperature for 120 min. Data were expressed as the mean ± SD by three independent experiments.

According to results from the AChE/BuChE inhibition and anti-inflammatory property, compound **3f** presented good dual AChE/BuChE inhibitory activity and significant anti-inflammatory potency. Thus, compound **3f** was selected to perform further investigation. In order to determine whether compound **3f** was a reversible hAChE and hBuChE inhibitor, the recovery of hAChE and hBuChE inhibitor inhibition after dilution were performed (Figure 5(A,B)^26^. For the hAChE reversibility of inhibition (Figure 5(A)), when donepezil, rivastigmine and compound 3f diluted to 0.1 × IC_50_, respectively, the hAChE activity increased to 10.6%, −0.8% and 1.7%, respectively. Moreover, the recovery of hAChE inhibitors inhibition after dilution was carried out with time monitoring. As shown in Figure 5(B), the hAChE activity restored to 103.6% by treating with 0.1× IC_50_ donepezil at 60 min, and treatment with 0.1 × IC_50_ rivastigmine and compound **3f**, hAChE activity restored to 88.7% and 94.7% at 120 min, respectively. The results showed that compound **3f** was a reversible hAChE inhibitor.

**Figure 6.**
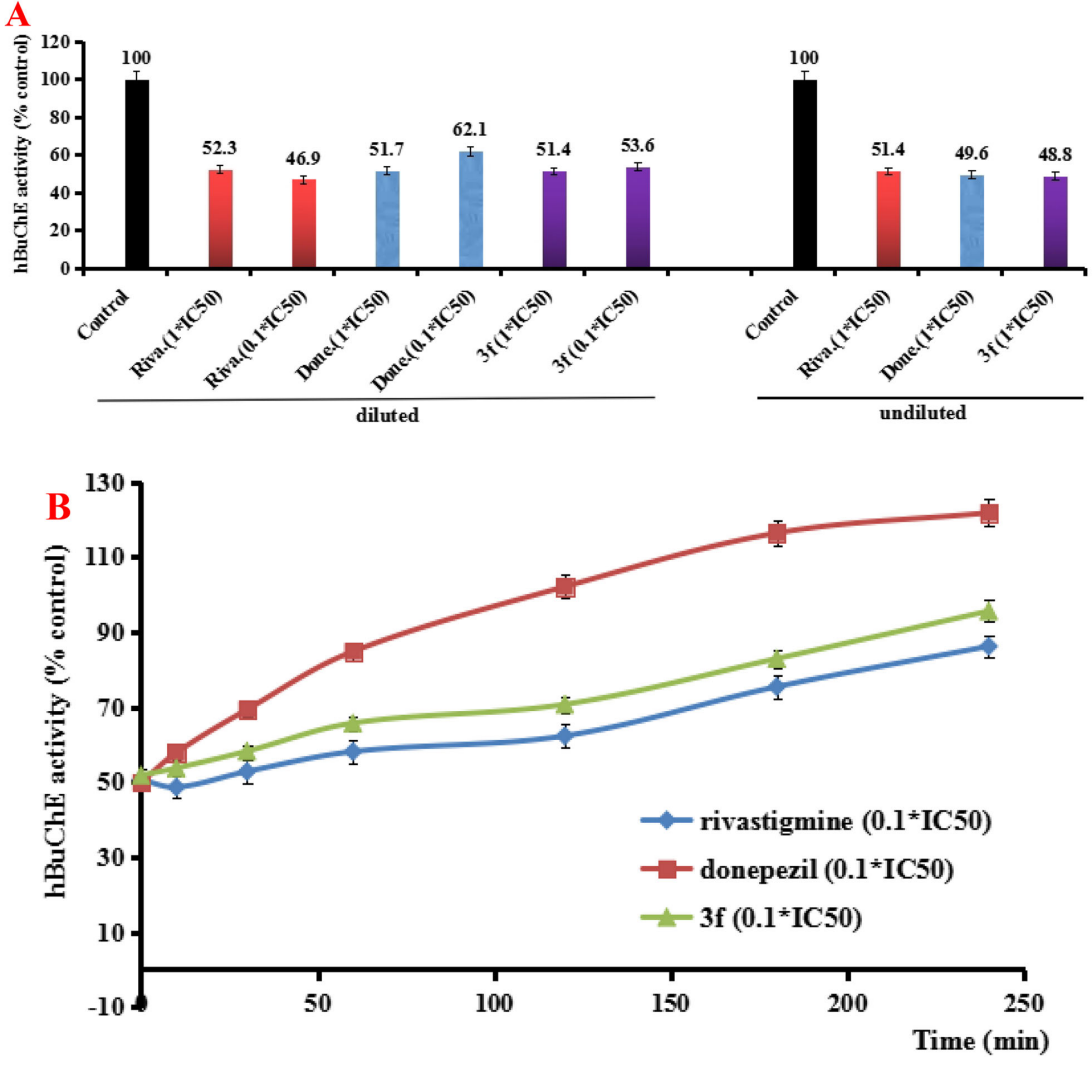
(A) hBuChE recovery after preincubation of compound **3f** diluted to 1× or 0.1 × IC_50_, compared to donepezil (done.) and rivastigmine (riva.) diluted, and undiluted inhibition. (B) hBuChE recovery of donepezil, rivastigmine and **3f** diluted to 0.1 × IC_50_, were monitored with time at room temperature for 240 min. Data were expressed as the mean ± SD by three independent experiments.

For the hBuChE reversibility of inhibition, as shown in Figure 6(A), compared to controls, the 0.1 × IC_50_ of donepezil, rivastigmine and compound **3f** increased the hBuChE activity to 8.3%, 1.2% and 3.1%, respectively. Furthermore, the recovery of hBuChE inhibitor inhibition after dilution with time monitoring was shown in Figure 6(B). When treating with 0.1× IC_50_ donepezil, rivastigmine and compound **3f** for 120 min, the hBuChE activity restored to 102.1%, 62.3% and 70.7%, respectively. As time went, the hBuChE activity restored to 86.2%, 121.8% and 95.6%, respectively at 240 min, suggesting that compound **3f** was a reversible hBuChE inhibitor. Therefore, the above results showed that compound **3f** was a reversible AChE and BChE inhibitor.

#### Enzyme kinetic study

**Figure 7.**
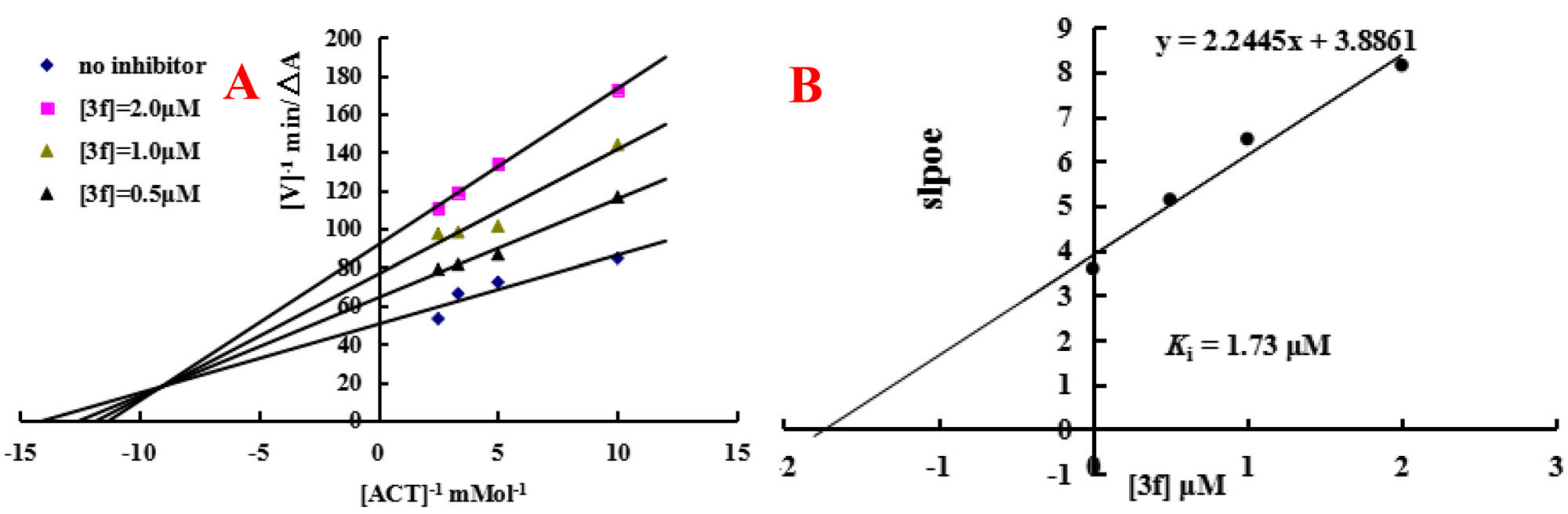
Enzyme kinetic study on the mechanism of *ee*AChE inhibition by compound **3f**. (A) Overlaid Lineweaver-Burk reciprocal plots of AChE initial velocity at increasing acetylthiocholine concentration in the absence and in the presence of **3f** were determined. (B) The plots of slope versus the concentration of **3f** for determining the inhibition constants *K*_i_.

In order to determine the mechanism of quinoline-*O*-carbamate derivatives, the representative compound **3f** was chose to perform Lineweaver-Burk double reciprocal plot by plotting the initial velocities of the substrate at y-axis and increasing concentrations substrate at x-axis^27^. As displayed in Figure 7(A), graphical analysis displayed both increasing slopes (decreased *V*_max_) and intercepts (higher *K*_m_) at increasing concentration of compound **3f**. As displayed in Figure 7(B), replots of the slope versus concentration of compound **3f** obtained an estimate of the inhibition constant and the *K*_i_ value was 1.73 µM. The results suggested that compound **3f** was a mixed-type of AChE inhibitor and was able to simultaneously bind both catalytic active site (CAS) and peripheral anionic site (PAS) of AChE.

#### Molecular modelling studies of 3f

**Figure 8.**
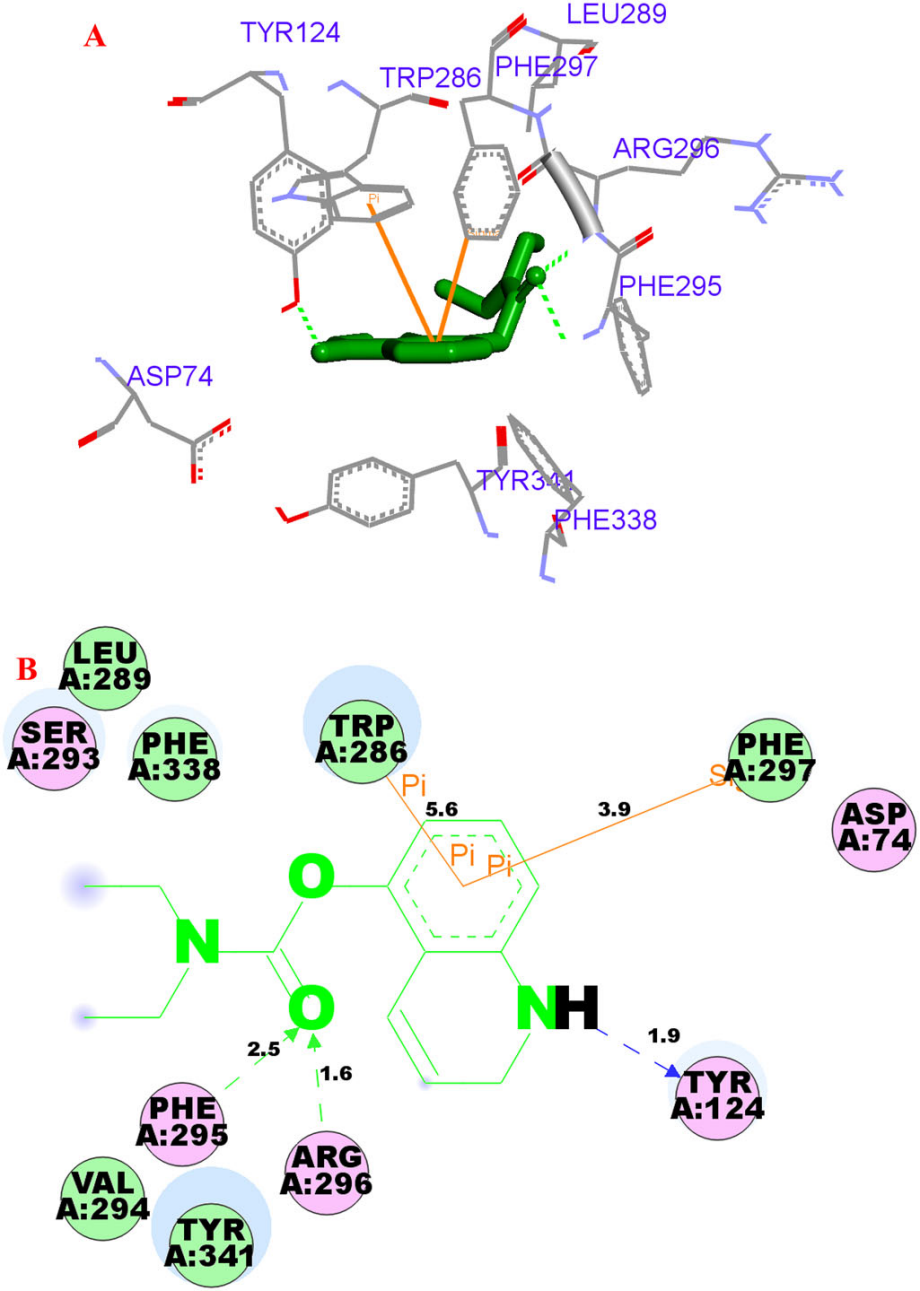
(A) Compound **3f** (green stick) acted on residues in the binding site of hAChE (PDB code: 4ey4). (B) D docking model of **3f** with hAChE.

The possible interacting mechanism of compound **3f** with hAChE (PDB code: 4ey4) and hBuChE (PDB code: 4tpk) was carried out using AUTODOCK 4.2 package, respectively^22^^,^^23^. It had been reported that amino acid residues Tyr72, Asp74, Trp86, Tyr124, Trp286, Tyr337, Phe295 and Phe297 were the key active site residues of hAChE^30^. As displayed in **3f**-hAChE complex (Figure 8), the carbonyl group interacted with Phe295 (2.5 Å) and Arg296 (1.6 Å) via two intramolecular hydrogen bonding, respectively. The polar H of quinoline interacted with Tyr124 (1.9 Å) *via* one intermolecular hydrogen bonding. The benzene ring of quinoline interacted with Trp286 (5.6 Å) and Phe297 (3.9 Å) *via* one π-π interaction and one σ-π interaction, respectively. Besides, some hydrophobic interactions were presented between compound **3f** and residues (such as Trp286, Tyr341, Phe338, Leu289, and Asp74). The above interactions offered a possible mechanism for its high AChE inhibitory activity.

**Figure 9.**
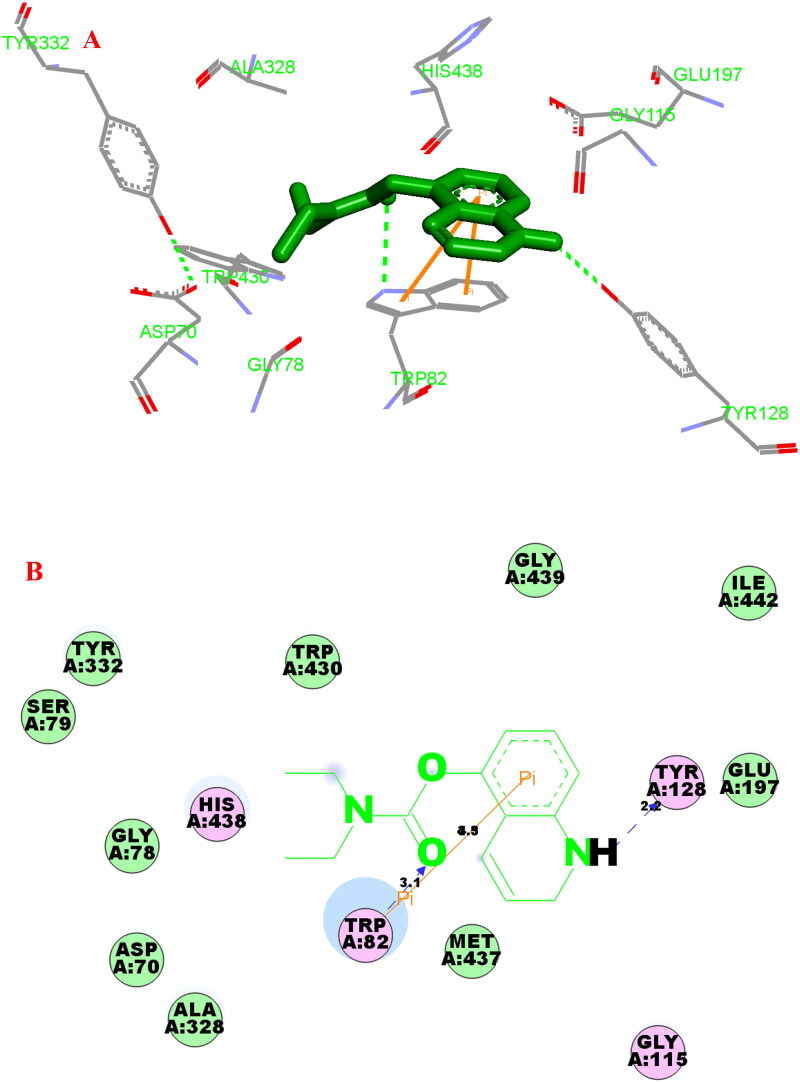
(A) Compound **3f** (green stick) acted on residues in the binding site of hBuChE (PDB code: 4tpk). (B) 2 D docking model of **3f** with hBuChE.

It had been verified that residues Trp82, His438, Val328, Phe329, Trp231, Val288, Gln119, Thr120 and Gly121 were considered to be an PDB entry 4tpk active site residues of hBuChE^31^. In the hBuChE −**3f** (Figure 9), the carbonyl group interacted with Trp82 via one intramolecular hydrogen bonding (3.1 Å). The polar H of quinoline interacted with Tyr128 *via* one intermolecular hydrogen bonding (2.2 Å). The benzene ring of quinoline interacted with Trp82 *via* two π-π interactions (3.3 Å and 4.5 Å). Moreover, some hydrophobic interactions were presented between compound **3f** and residues (such as Trp82, Tyr332, Glu197, Gly115, Tyr128, Trp430, His438 and Ala328). Thus, the above observed phenomenon could provide a reasonable binding mechanism for potent BChE inhibitory activity of **3f**.

#### Molecular dynamics (MD) simulations

**Figure 10.**
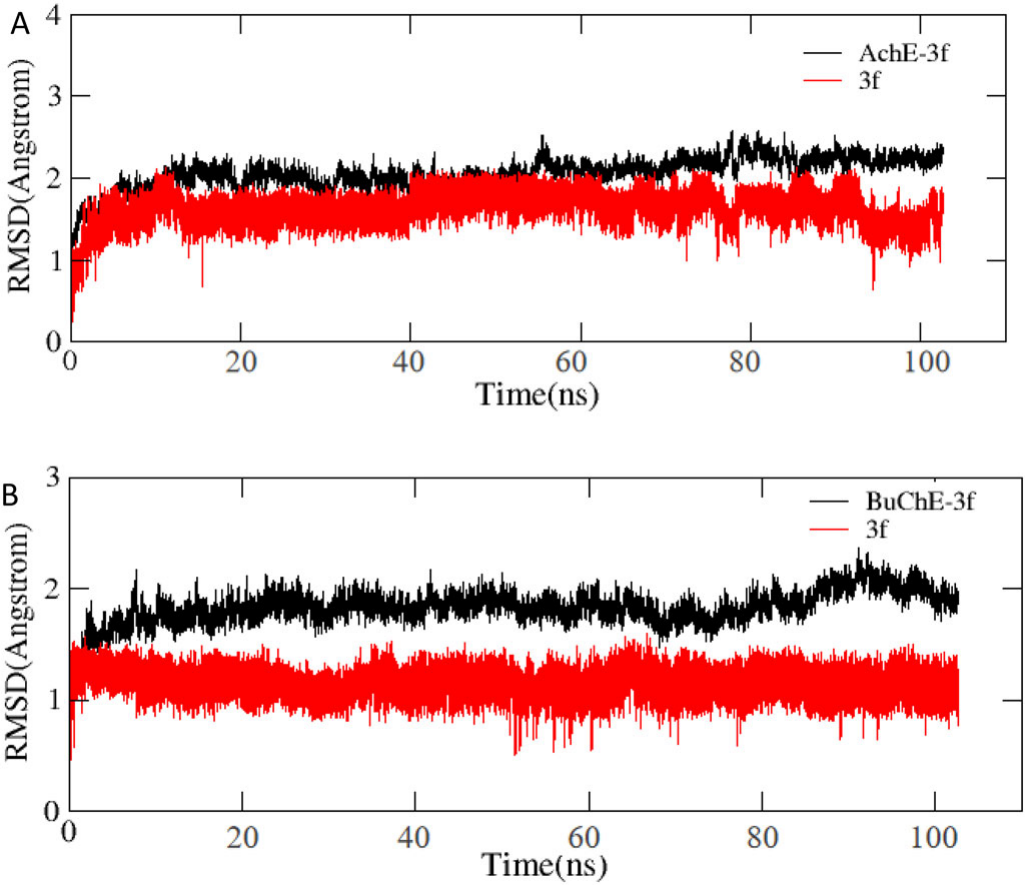
(A) RMSD analysis of compound **3f** with hAChE (PDB code: 4ey4) (black stick); (B) RMSD analysis of compound **3f** with hBuChE (PDB code: 4tpk) (black stick).

**Figure 11.**
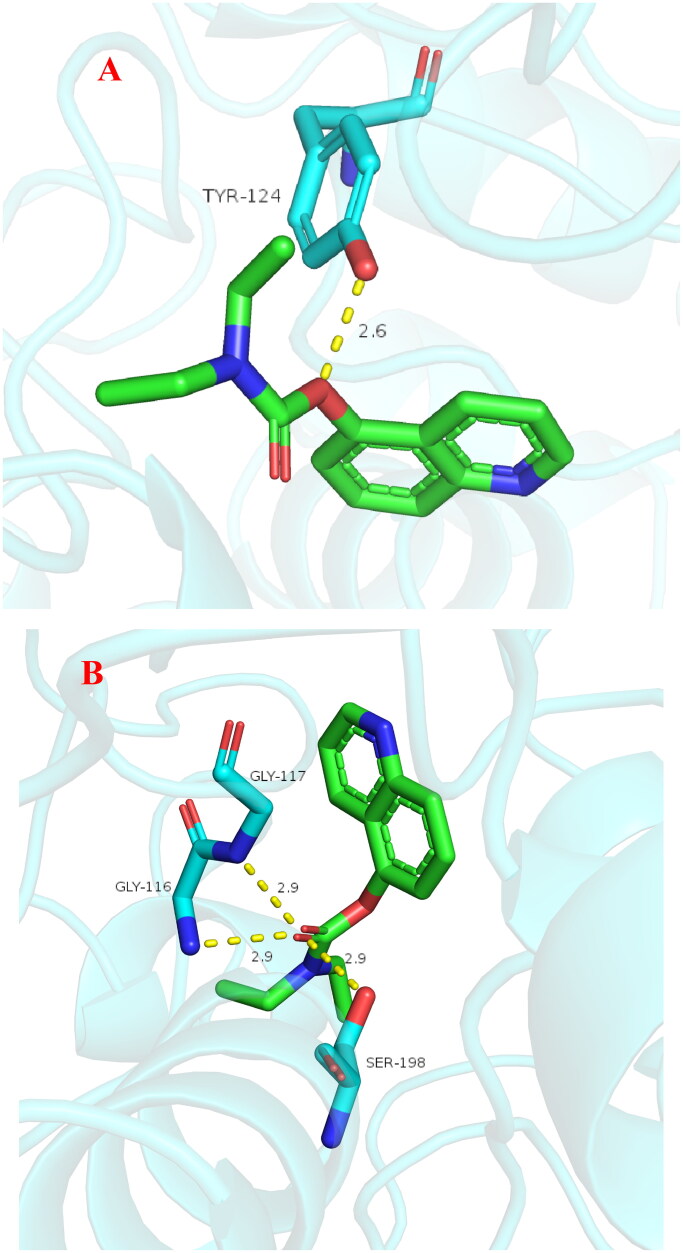
(A) The docking model for **3f** into protein crystal structure of hAChE (PDB code: 4ey4). (B) The docking model for **3f** into protein crystal structure of hBuChE (PDB code: 4tpk).

The stability of docked binding pose of the compound **3f**-AChE complex and **3f**-BuChE were analysed by molecular dynamics simulation analysis using Amber 16^24^. Figure 10 shows that the root means square deviations (RMSD) analysis of compound **3f** with the amino acid residues of AChE and BuChE, respectively. The results demonstrated that RMSDs of all the replicas for the six simulated systems presented relatively stable fluctuations after 50 ns of the MSMD simulations, suggesting that the six simulated systems basically reach equilibrium. The binding free energies of compound **3f** towards hAChE and hBuChE calculated using MM-PBSA were displayed in Table 2, and have values of −30.59 and −37.65 kcal/mol, respectively, which were mainly contributed by Van der Waals forces, electrostatic interactions and non-polar solvation energies^24^. Furthermore, Figure 1**1** shows the key residues and interaction modes of compound **3f** with hAChE and hBuChE, respectively. In Figure 11(A), the *O* atom *O*-carbamate fragment formed one intermolecular hydrogen bonding with the key amino acid Tyr124 (2.6 Å). Figure 11(B) displays the interactions modes of **3f** with hBuChE, the carbonyl group of carbamate fragment interacted with key amino acid residue Gly116 (2.9 Å), Gly117 (2.9 Å) and Ser198 (2.9 Å) *via* one intermolecular hydrogen bonding interactions, respectively.

**Table 2.** The binding free energy and components of **3f**-AChE and **3f-**BuChE (kcal/mol).

Terms	Δ*E_vdw_*	Δ*E_eel_*	Δ*E_egb_*	Δ*E_esurf_*	Δ*G_gas_*	Δ*G_solv_*	Δ*G_bind_*
**3f-**AChE	−35.45 (0.06)	−18.18 (0.09)	24.92 (0.07)	−3.07 (0.002)	−53.64 (0.08)	21.85 (0.07)	−31.79 (0.08)
**3f-**BuChE	−38.27 (0.05)	−21.49 (0.06)	25.18 (0.05)	−3.12 (0.003)	−59.76 (0.07)	22.06 (0.05)	−37.70 (0.06)

#### Neuroprotective effect

**Figure 12.**
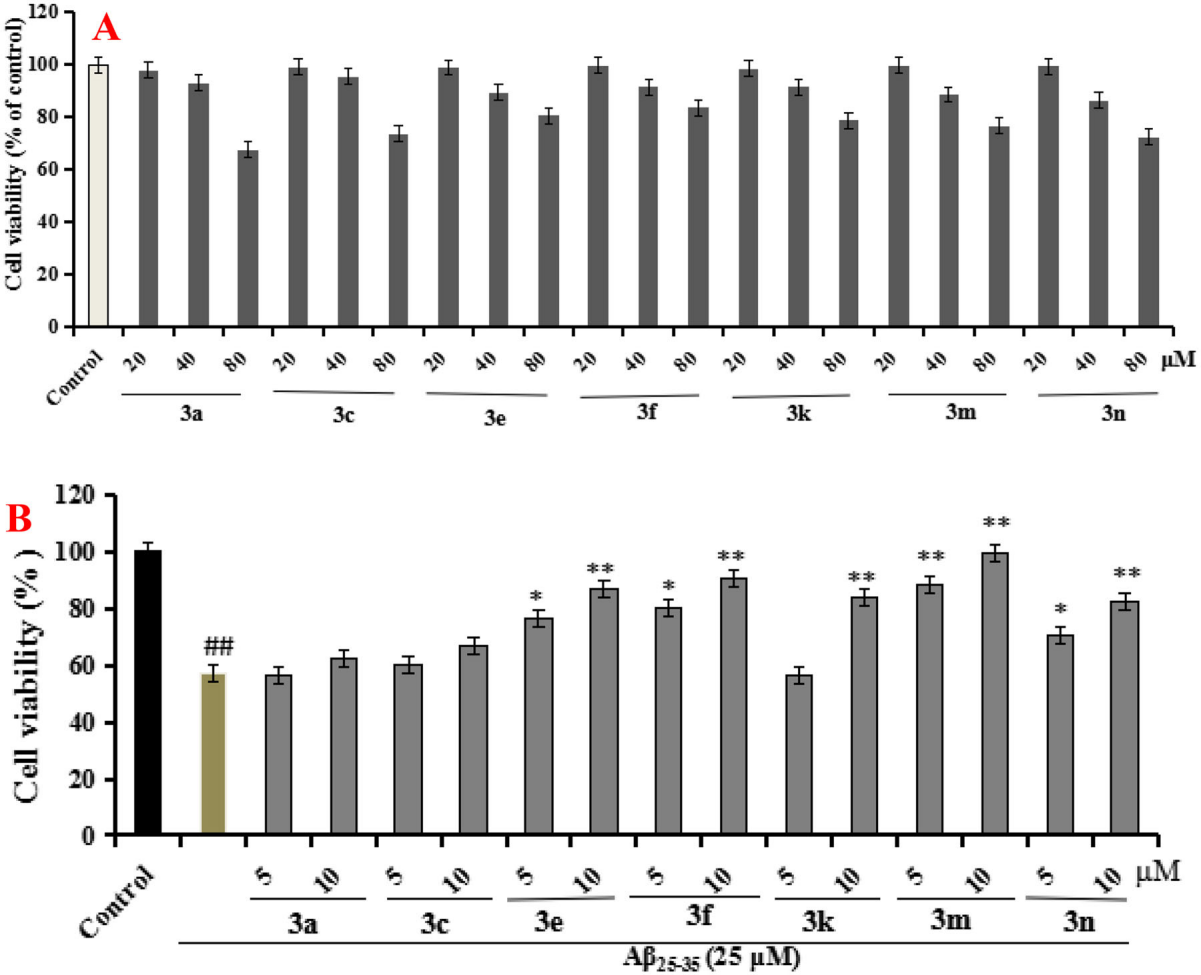
Cell viability was tested by MTT assay. (A) Cytotoxicity of compounds **3a**, **3c**, **3e**, **3f**, **3k**, **3m**, and **3n** on PC12 cells. (B) Attenuation of A*β*_25-35_-induced PC12 cell injury by compounds **3a**, **3c**, **3e**, **3f**, **3k**, **3m**, and **3n**. values were expressed as mean ± SD by three independent experiments. ##*p* < 0.01 vs control; **p < 0.01, *p < 0.05 vs A*β*_25-35_ group.

The neuroprotective effect of compounds **3a**, **3c**, **3e**, **3f**, **3k**, **3m**, and **3n** on A*β*_25–35_-induced PC12 cells injury were evaluated using CCK-8 assay^32^. As presented in Figure 12(A), compounds **3a**, **3c**, **3e**, **3f**, **3k**, **3m**, and **3n** did not show any cytotoxicity at concentration below 40 µM, indicating a widely safe range. Furthermore, as shown in Figure 12(B), when PC12 cells were exposed to 25 µM Aβ_25-35_, the cell viability sharply declined to 57.3% (*p*** **<** **0.01) compared with untreated group. When the PC12 cells was treated with compounds **3a**, **3c**, **3e**, **3f**, **3k**, **3m**, and **3n**, respectively, the results showed that compounds **3a** and **3c** with carbamate fragment at 4 positin of quinoline did not show significant neuroprotective effects on A*β*_25-35_-induced PC12 cells injury, compound **3k** with carbamate fragment at 6 positin of quinoline showed significant neuroprotective effects at 10 µM, compounds **3e** and **3f** with carbamate fragment at 5 positin of quinoline displayed remarkable neuroprotective effects at 5 and 10 µM, respectively, compounds **3m** and **3n** with carbamate fragment at 8 positin of quinoline showed significant neuroprotective effects at 5 and 10 µM, respectively.

#### Stability studies of 3f

**Figure 13.**
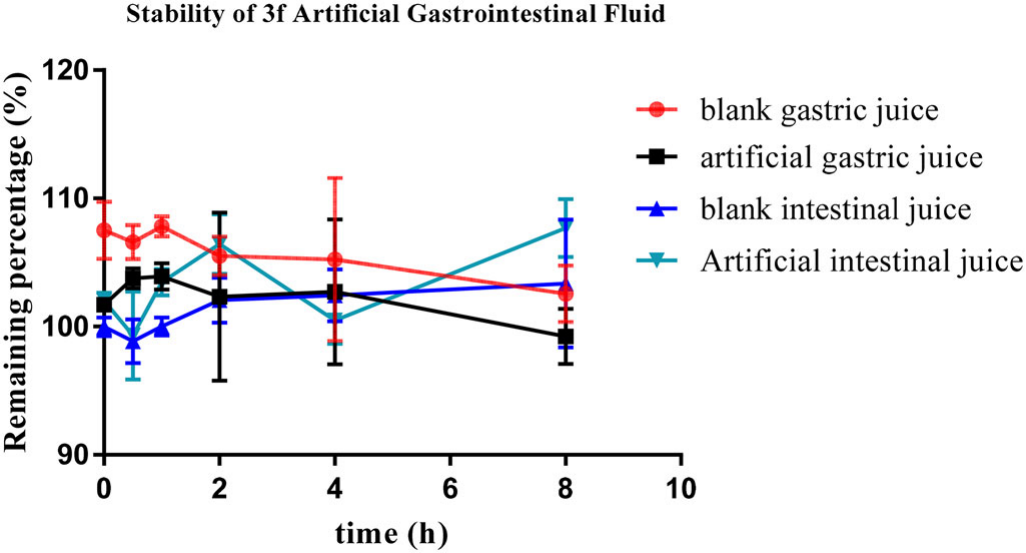
Stability of compound **3f** in artificial gastrointestinal fluids (*n* = 3).

Compound **3f** was selected to investigate the stabilities by artificial gastrointestinal conditions and (human and rat) liver microsomes^33^. As displayed in Figure 13, the remaining amount of compound **3f** in blank gastric fluid, blank intestinal fluid, artificial gastric fluid and artificial intestinal fluid was 102.6%, 103.4%, 99.2% and 107.7%, respectively, after 8 h incubation, suggesting that compound **3f** was stable in both artificial intestinal and gastric fluids.

**Figure 14.**
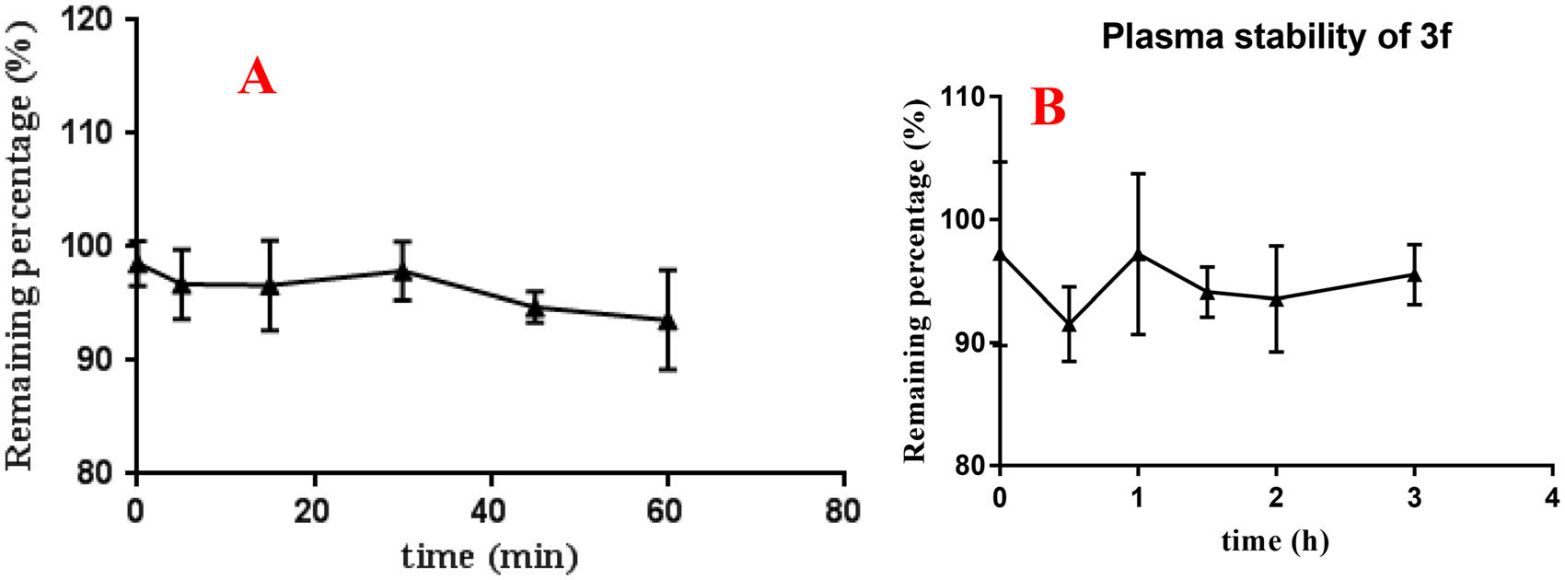
(A) rat liver microsomes stabilities of compound **3f** (*n* = 3); (B) the plasma stability of compound **3f** (*n* = 3).

The stability of compound **3f** was also performed using rat liver microsomes assay. According to the results from Figure 14(A), after 60 min incubation, the remaining amount of **3f** in rat liver microsomes was 104.4%. Futher, the plasma stability of compound **3f** was also performed using rat plasma. As displayed in Figure 14(B), the remaining amount of **3f** was 95.5% after 3 h incubation in rat plasma.

The above results showed that compound **3f** presented good stabilities in artificial gastrointestinal fluids and moderate stabilities in liver microsomes.

#### Effect of 3f on AlCl_3_-induced zebrafish AD

AlCl_3_-induced zebrafish AD model has been widely used for high throughput screening of potential novel anti-AD leading compounds^34^^,^^35^.

**Figure 15.**
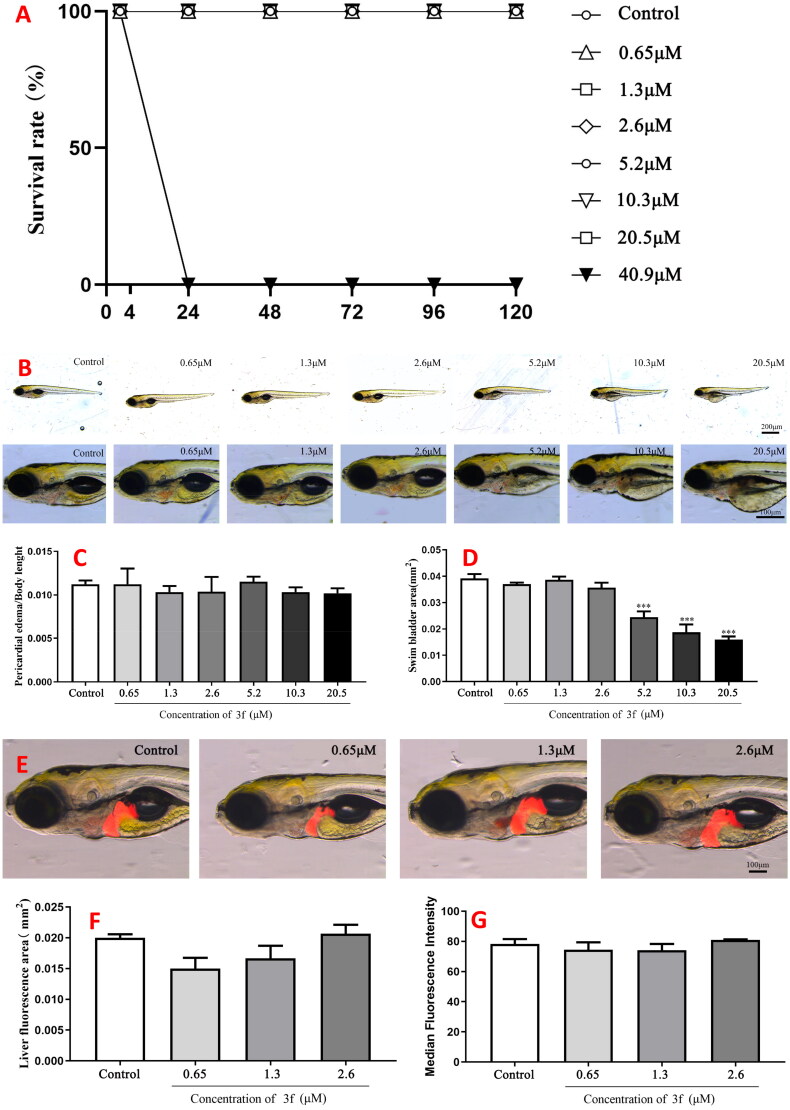
The acute toxicity of compound **10c**. (A) The percentage survival (%); (B) morphologic change; (C) changes of pericardial edema/body length; (D) changes of swim bladder area; (E) changes in the liver; (F) changes of liver fluorescence area; (G) changes of median fluorescence intensity.

**Figure 16.**
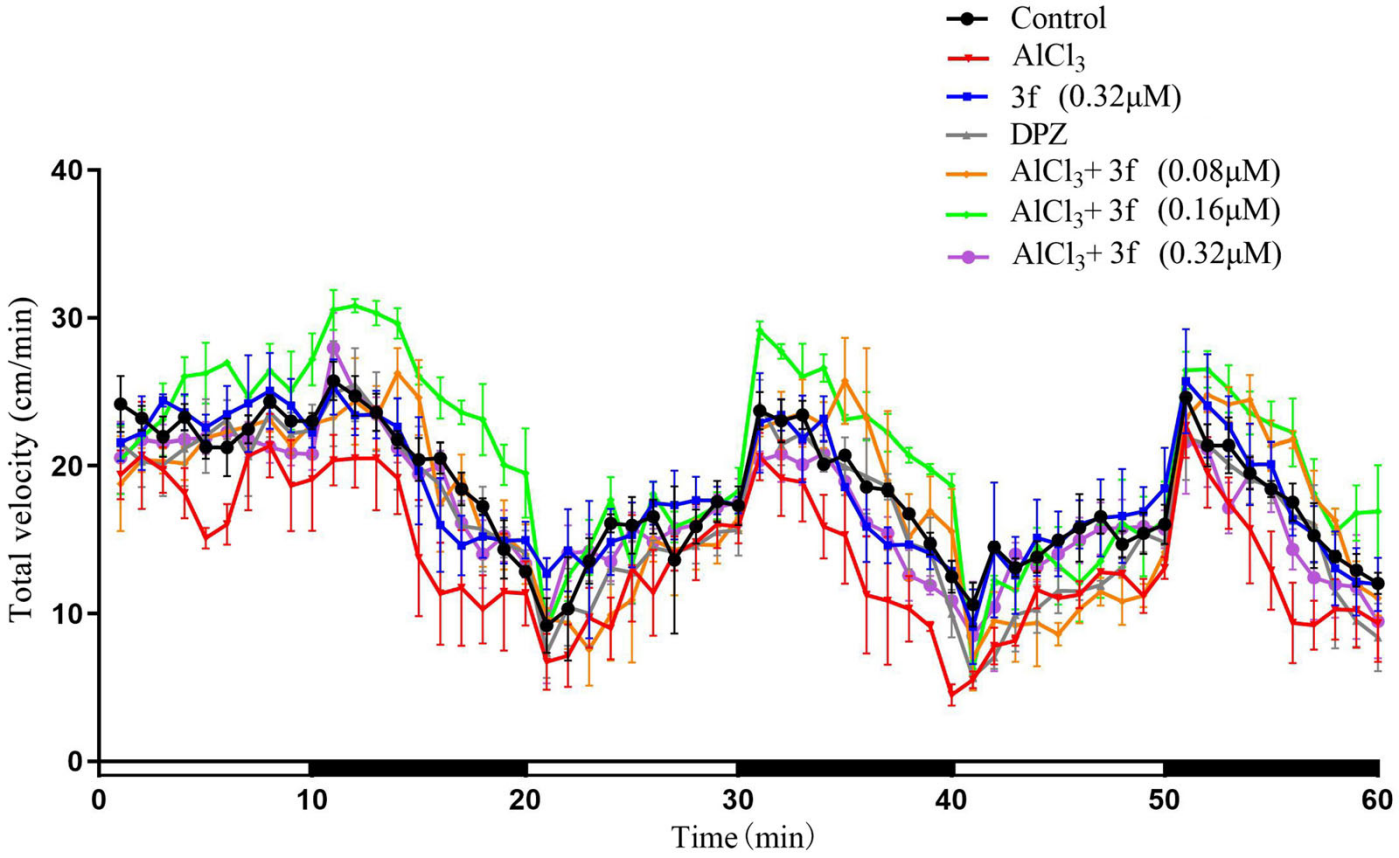
The total velocity of compound **3f** on AlCl_3_-induced zebrafish AD model (*n* = 60). And the white and dark stripes on x-axis represent periods of light and dark, respectively.

1. Acute toxicity. Firstly, the safety profile of compound **3f** were investigated with seven concentrations (0.65, 1.3, 2.6, 5.2, 10.3, 20.5 and 40.9 µM). As displayed in Figure 15(A), when the concentration increased to 40.9 µM, the percentage survival was 100% after 4h of post fertilisation (hpf), while all the zebrafish were dead for 24 hpf. When the concentration under 20.5 µM, the survival rate of other concentrations were 100% during 120 hpf. The results showed that the maximum non-lethal concentration (MNLC) of **3f** was 20.5 µM. And based on the MNLC, six concentrations of **3f** (0.65, 1.3, 2.6, 5.2, 10.3 and 20.5 µM) were selected to further investigate the acute toxicity. As shown in Figure 15(B–D), the pericardial oedema/body length did not produce significant change at five concentrations of **3f**, while the swim bladder area significantly decreased after treating with 5.2 µM **3f**. So, three concentrations of **3f** (0.65, 1.3 and 2.6 µM) were selected to further investigate the liver toxicity. As listed in Figure 15(E–G), both the liver florescence area and median fluorescence intensity did not produce obvious change at three concentrations, suggesting that compound **3f** was safe at 2.6 µM.
2. *Effects of* ***3f*** *on AlCl_3_-Induced Zebrafish AD Model.* Based on the above results, compound **3f** with three concentrations (0.08, 0.16 and 0.32 µM) were performed to evaluate the dyskinesia and reaction ././Lenovo/AppData/Local/youdao/dict/Application/7.5.2.0/resultui/dict/?keyword = capacity of AlCl_3_-induced zebrafish AD. The total velocity were shown in Figure 16, compound **3f** at dose of 0.16 µM could improve AlCl_3_-induced zebrafish AD model compared with donepezil.

**Figure 17.**
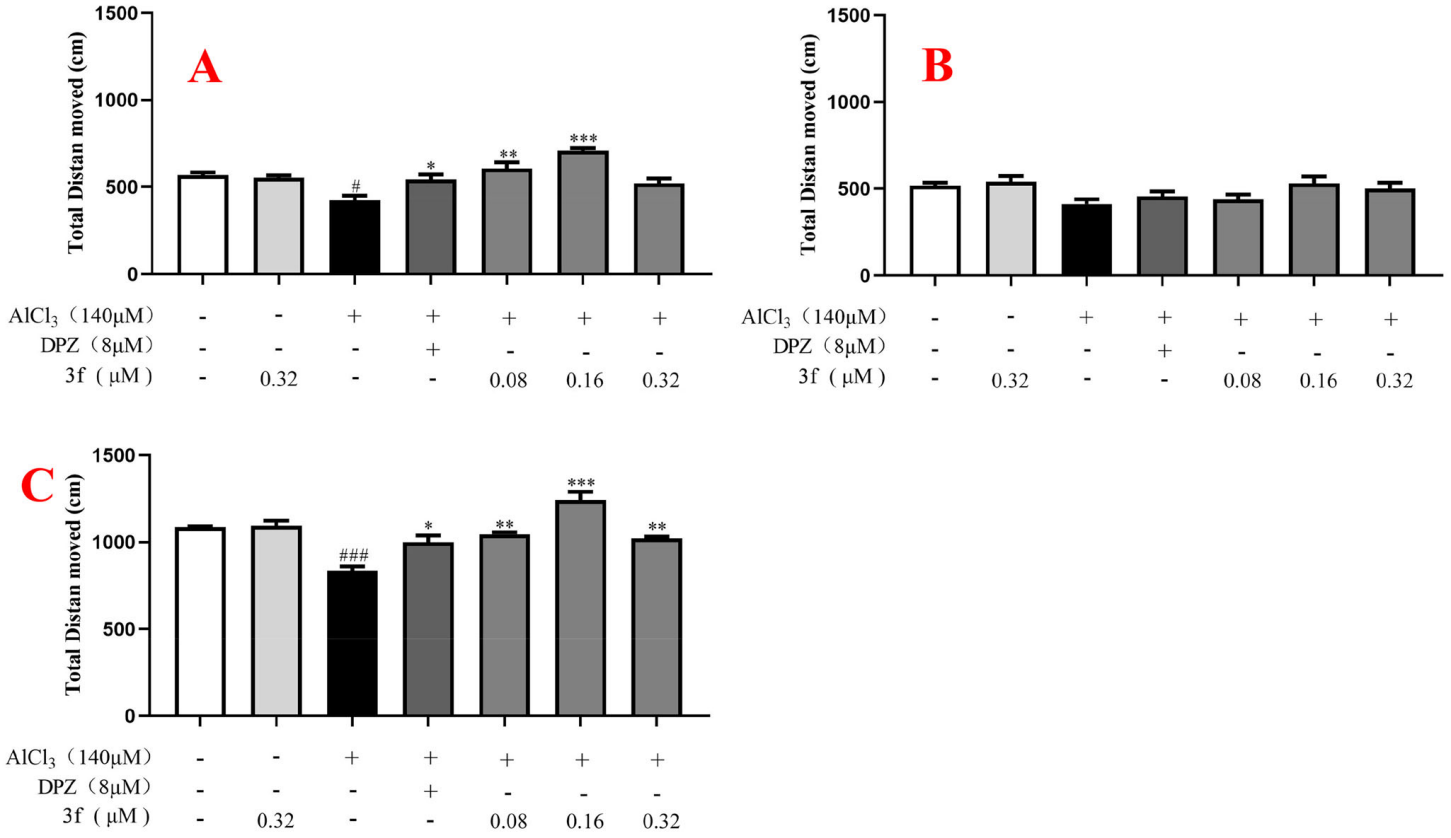
The total distance moved of compound **3f** on AlCl_3_-induced zebrafish AD model. (A) Under the dark environment; (B) Under the light environment; (C) Under alternating dark light stimuli condition. ^#^*p*** **<** **0.05 and ^###^*p*** **<** **0.001 *vs* untreated group. *p** **<** **0.05, **p** **<** **0.01 and **p** **<** **0.001 *vs* model group.

More specifically, for the distance moved, in the dark environment (Figure 17(A)), when zebrafish were treated with 140 µM AlCl_3_, the distance moved significantly declined to 447.4 cm (*p* < 0.05) compared with untreated group (582.9 cm). When treatment with 8 µM donepezil, the distance moved increased to 543.8 cm (*p* < 0.05). When treatment with compound **3f** (0.08, 0.16 and 0.32 µM), the distance moved was 607.3 cm (*p* < 0.01), 709.4 cm (*p* < 0.001) and 521.7 cm, respectively. While, in the light environment (Figure 17(B)), when zebrafish were exposed to 140 µM AlCl_3_, the distance did not show statistical significance between the untreated group, model group, and tested compound group. Furthermore, under alternating dark light stimuli condition (Figure 17(C)), when treatment with compound **3f** (0.08, 0.16 and 0.32 µM), the distance moved was 1029.2 cm (*p* < 0.01), 1241.5 cm (*p* < 0.001) and 1022.8 cm (*p* < 0.01), respectively, which was better than 8 µM donepezil (999.4 cm). The results showed that in the dark or alternating dark light stimuli condition, 8 µM donepezil could improve distance moved in the AlCl_3_-induced zebrafish AD model. Moreover, under the dark condition, light condition, and alternating dark light condition, respectively, 0.16 µM compound **3f** presented the best distance moved.

**Figure 18.**
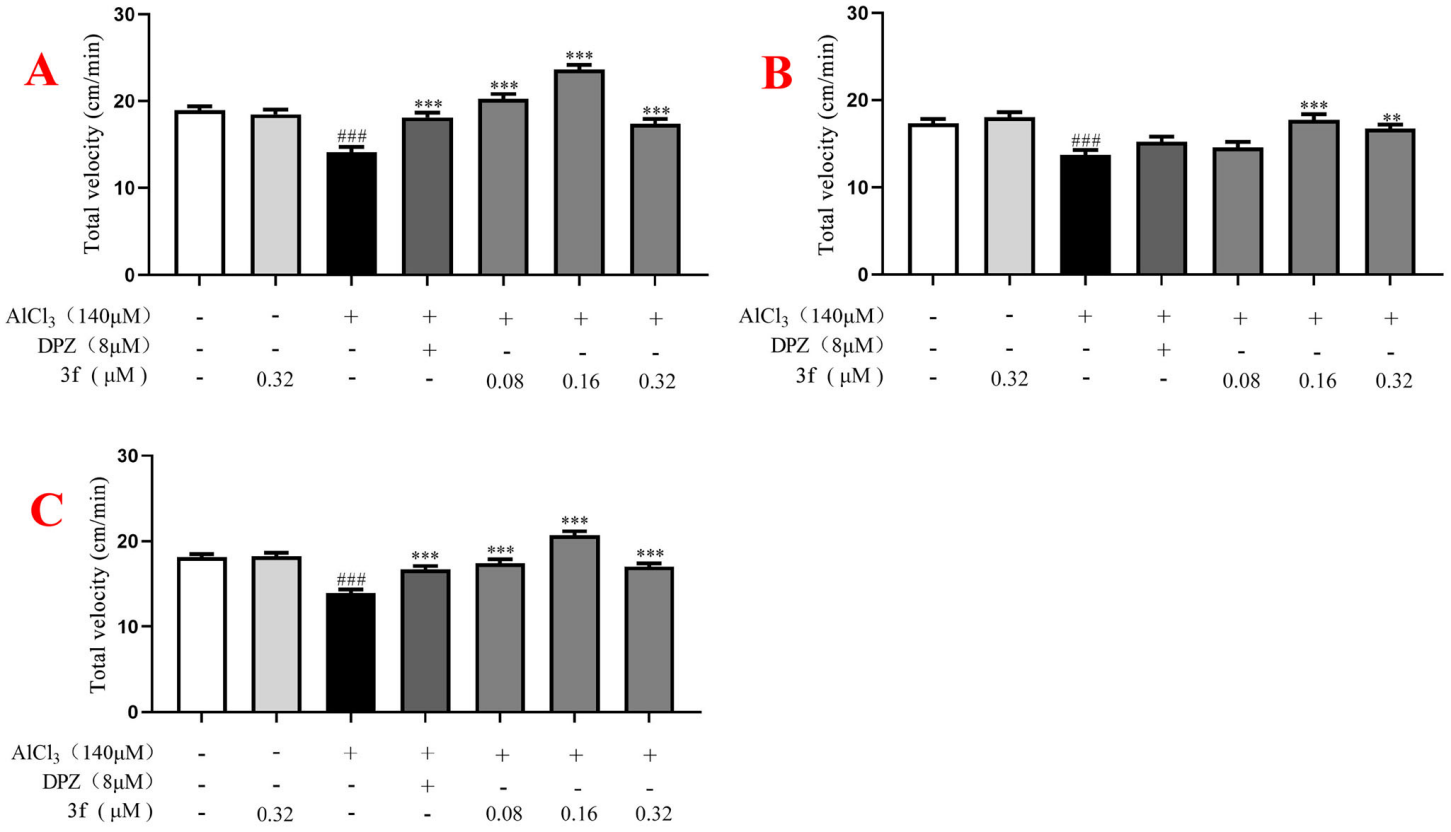
The total velocity of compound **3f** on AlCl_3_-induced zebrafish AD model. (A) Under the dark environment; (B) under the light environment; (C) under alternating dark light stimuli condition. ^#^*p*** **<** **0.05 and ^###^*p*** **<** **0.001 *vs* untreated group. *p** **<** **0.05, **p** **<** **0.01 and **p** **<** **0.001 *vs* model group.

For the velocity, in the dark environment (Figure 18(A)), when zebrafish were treated with 140 µM AlCl_3_, the velocity significantly declined to 13.8 cm/min (*p* < 0.001) compared with untreated group (19.1 cm/min). When treatment with 8 µM donepezil, the velocity increased to 18.5 cm/min (*p* < 0.001). When treatment with compound **3f** (0.08, 0.16 and 0.32 µM), the velocity was 21.3 cm/min (*p* < 0.001), 24.7 cm/min (*p* < 0.001), and 17.3 cm/min (*p* < 0.001), respectively. In the light condition (Figure 18(B)**)**, when treatment with compound **3f** (0.08, 0.16 and 0.32 µM), the velocity were 14.7 cm/min, 19.3 cm/min (*p* < 0.001) and 17.6 cm/min (*p* < 0.01), respectively. In alternating dark light stimuli condition (Figure 18(C)), when treatment with compound **3f** (0.08, 0.16 and 0.32 µM), the velocity were 17.5 cm/min (*p* < 0.001), 22.3 cm/min (*p* < 0.001) and 17.0 cm/min (*p* < 0.001), respectively, which was better than donepezil (16.7 cm/min). The results showed that 8 µM donepezil significantly improved velocity in the AlCl_3_-induced zebrafish AD model under dark or alternating dark light stimuli condition. Moreover, under the dark, light and alternating dark light stimuli condition, respectively, 0.16 µM compound **3f** presented the best velocity.

To sum up, compound **3f** could significantly improve dyskinesia and reaction ././Lenovo/AppData/Local/youdao/dict/Application/7.5.2.0/resultui/dict/?keyword = capacity of AlCl_3_-induced zebrafish AD model, which was better than donepezil, and the optimal dose was 0.16 µM.

**Figure 19.**
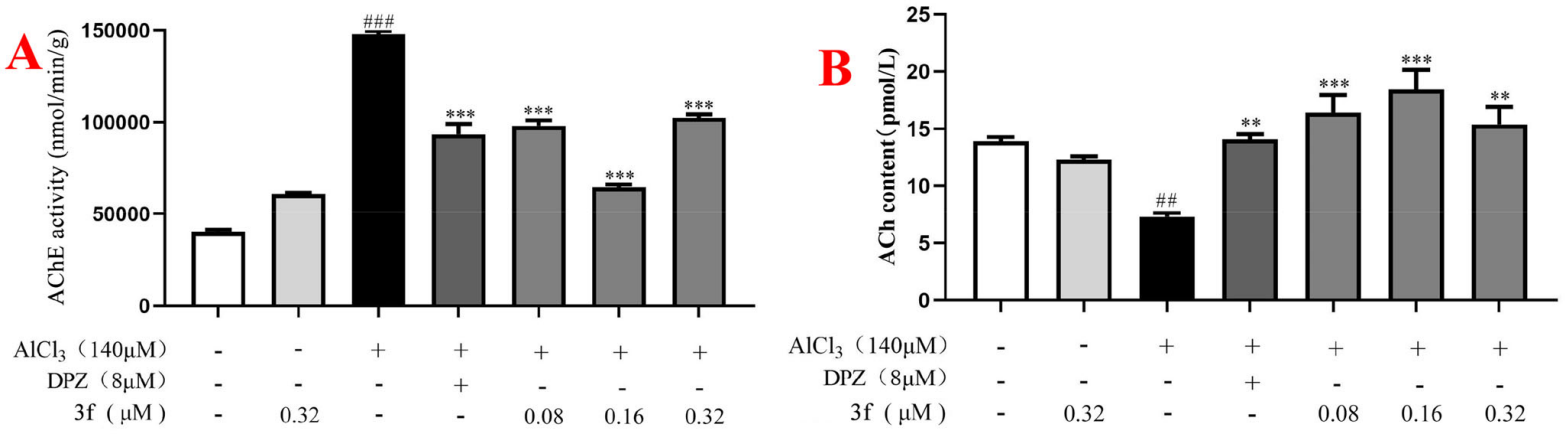
(A) The AChE activity of **3f** on AlCl_3_-induced zebrafish AD model. (B) The ACh level of **3f** on AlCl_3_-induced zebrafish AD model. ^##^*p*** **<** **0.01 and ^###^*p*** **<** **0.001 *vs* untreated group. **p** **<** **0.01 and **p** **<** **0.001 *vs* model group.

3.Mechanisms of compound **3f** on AlCl_3_-induced AD in zebrafish model. After the behavioural experiments, the 168hpf zebrafish were killed and ground, the tissue were used to test the AChE activity and ACh levels. As shown in Figure 19(A), when zebrafish were exposed to 140 µM AlCl_3_, the AChE activity sharply increased to 147908.7 nmol/min/g (p < 0.001) compared with untreated zebrafish (40018.7 nmol/min/g). When AD zebrafish model were treated with 8 µM donepezil, the AChE activity significantly decreased to 93540.7 nmol/min/g (p < 0.001). When treatment with compound **3f** (0.08, 0.16 and 0.32 µM), the AChE activity were 98095.7 nmol/min/g (p < 0.001), 64663.2 nmol/min/g (p < 0.001) and 102433.6 nmol/min/g (p < 0.001), respectively. Furthermore, Figure 19(B) shows that when zebrafish were treated with 140 µM AlCl_3_, the ACh activity sharply decreased to 7.3 (p < 0.01) compared with untreated group (13.9 pmol/L). When treatment with 8 µM donepezil, the ACh activity significantly increased to 14.0 pmol/L (p < 0.01). When treatment with compound **3f** (0.08, 0.16 and 0.32 µM), the ACh activity were 16.4 pmol/L (p < 0.001), 18.4 pmol/L (p < 0.001) and 15.4 pmol/L (p < 0.01), respectively. The results suggested that compound **3f** could improve the levels of ACh in AD zebrafish model by inhibiting AChE activity, and the optimal dose of compound **3f** was 0.16 µM.

## Conclusion

Alzheimer’s disease (AD) was a chronic, progressive brain neurodegenerative disorder. Up to now, there was no effective drug to halt or reverse the progress of AD. Given the complex pathogenesis of AD, the MTDLs strategy was considered as the promising approach. Herein, a series of novel quinoline-*O*-carbamate derivatives was rationally designed for treating AD by fusing quinoline and rivastigmine. The synthesised target compounds were synthesised and evaluated by AChE/BuChE inhibition and anti-inflammatory property. The *in vitro* biological activities revealed that compound **3f** was a reversible dual *ee*AChE/eqBuChE inhibitor with IC_50_ values of 1.3 µM and 0.81 µM, respectively. Compound **3f** also showed good anti-inflammatory property by declining the production of inflammatory mediators IL-6, IL-1β and NO. In addition, compound **3f** presented significant neuroprotective effect on A*β*_1-42_-induced PC12 cell injury. Moreover, compound **3f** possessed favourable stabilities in artificial gastrointestinal fluids, liver microsomes *in vitro* and plasma. Furthermore, compound **3f** significantly improved dyskinesia and reaction ././Lenovo/AppData/Local/youdao/dict/Application/7.5.2.0/resultui/dict/?keyword = capacity of AlCl_3_-induced zebrafish AD model by increasing the level of ACh. Therefore, compound **3f** was a promising multifunctional agent for the treatment of AD, deserving for further investigation.

## Experimental section

### Chemistry

Unless otherwise noted, all the chemicals and solvents were obtained from Shanghai Titan Scientific Co.,Ltd. and Sigma-Aldrich, and were used without further purification. All the reactions were monitored by thin-layer chromatography (TLC) using silica gel GF254 plates, and crude products were purified by column chromatography using silica gel (230–400 mesh) purchased from Qingdao Haiyang Chemical Co. Ltd. (China). The purity of the target compounds was determined by high-performance liquid chromatography (HPLC) analysis to be over 96%. HPLC analysis was carried out on a Waters X-Bridge C18 column (4.6 mm × 150 mm, 5 µm) at a flow ratio of 0.8 ml/min. The ^1^H NMR and ^13^C NMR spectra of the synthetic compounds were recorded using TMS as the internal standard on a Varian INOVA at 400 and 100 MHz, respectively. The high-resolution mass spectra was obtained using a Waters Xevo G2-XS-Qtof mass spectrometer.

#### General procedures for the synthesis of quinoline-O-carbamate derivatives

To a solution of the starting material **1** (4-hydroxyquinoline **1a**, 5-hydroxyquinoline **1b**, 6-hydroxyquinoline **1c**, and 8-hydroxyquinoline **1d**, respectively) (1.0 mmol), and anhydrous K_2_CO_3_ (1.7 mmol) in anhydrous CH_3_CN (6 ml), *N,N*-disubstituted carbamoyl chlorides **2a–2e** (1.5 mmol) was added dropwise in ice-water bath. The mixture was naturally heated to room temperature and then heated to 65 °C for 6–10 h. After the reaction completed, the solvent was evaporated under reduced pressure. 20 ml water was added to the residue, which was further extracted with CH_2_Cl_2_ (2 × 20 ml). The combined organic phases were washed by saturated aqueous 50 ml NaCl, dried over anhydrous Na_2_SO_4_ and filtered. The solvent was evaporated to dryness under reduced pressure to obtain the crude product, which was further purified on a silica gel chromatography using petroleum ether/acetone (50:1) as eluent to afford the desired target derivatives **3a∼3q**.

#### Quinolin-4-yl dimethylcarbamate (3a)

The starting material 4-hydroxyquinoline **1a** was treated with dimethylcarbamoyl chloride **2a** to get target derivative **3a**. Light yellow oil, yield 45% (97 mg) and 97.8% HPLC purity. ^1^H NMR 8.88 (d, *J*** **=** **4.8 Hz, 1H, Ar-H), 8.12 (d, *J*** **=** **8.4 Hz, 1H, Ar-H), 8.01 (d, *J*** **=** **8.4 Hz, 1H, Ar-H), 7.72 (d, *J*** **=** **5.6 Hz, 1H, Ar-H), 7.55 (t, *J*** **=** **8.0 Hz, 1H, Ar-H), 7.38 (d, *J*** **=** **5.2 Hz, 1H, Ar-H), 3.24 (s, 3H, NCH_3_), 3.07 (s, 3H, NCH_3_). HR-ESI-MS: Calcd. for C_12_H_12_N_2_O_2_ [M + H]^+^: 217.0932, found: 217.0971.

#### Quinolin-4-yl diethylcarbamate (3 b)

The starting material 4-hydroxyquinoline **1a** was treated with diethylcarbamoyl chloride **2b** to get target derivative **3b**. Light yellow oil, yield 41% (100 mg) and 98.4% HPLC purity. ^1^H NMR 8.89 (d, *J*** **=** **4.8 Hz, 1H, Ar-H), 8.12 (d, *J*** **=** **8.4 Hz, 1H, Ar-H), 8.01 (d, *J*** **=** **8.4 Hz, 1H, Ar-H), 7.73 (d, *J* = 8.0 Hz, 1H, Ar-H), 7.56 (t, *J*** **=** **8.0 Hz, 1H, Ar-H), 7.39 (d, *J* = 4.8 Hz, 1H, Ar-H), 3.59 (q, *J*** **=** **7.2 Hz, 2H, NCH_2_), 3.46 (q, *J*** **=** **7.2 Hz, 2H, NCH_2_), 1.36 (t, *J*** **=** **7.2 Hz, 3H, CH_3_), 1.26 (t, *J*** **=** **7.2 Hz, 3H, CH_3_). ^13^C NMR (100 MHz, CDCl_3_) δ 154.9 (=C-O), 152.5 (C = O), 150.9 (C = N), 149.9 (=C-N), 129.8 (Ph-C), 129.4 (Ph-C), 126.6 (Ph-C), 122.8 (Ph-C), 121.3 (Ph-C), 112.2 (=C), 42.6 (NCH_2_), 42.3 (NCH_2_), 14.5 (CH_3_), 13.3 (CH_3_). HR-ESI-MS: Calcd. for C_13_H_14_N_2_O_2_ [M + H]^+^: 245.1245, found: 245.1284.

#### Quinolin-4-yl ethyl(methyl)carbamate (3c)

The starting material 4-hydroxyquinoline **1a** was treated with *N*-ethyl-*N*-methylcarbamoyl chloride **2c** to get target derivative **3c**. Light yellow oil, yield 39% (90 mg) and 97.0% HPLC purity. ^1^H NMR 8.89 (d, *J*** **=** **4.8 Hz, 1H, Ar-H), 8.12 (d, *J*** **=** **8.4 Hz, 1H, Ar-H), 8.01 (t, *J*** **=** **7.6 Hz, 1H, Ar-H), 7.72 (d, *J* = 8.0 Hz, 1H, Ar-H), 7.55 (t, *J*** **=** **7.6 Hz, 1H, Ar-H), 7.39 (d, *J* = 7.6 Hz, 1H, Ar-H), 3.60 + 3.46 (q, *J*** **=** **7.2 Hz, 2H, NCH_2_), 3.21 + 3.05 (s, 3H, NCH_3_), 1.34 + 1.24 (t, *J*** **=** **7.2 Hz, 3H, CH_3_). ^13^C NMR (100 MHz, CDCl_3_) δ 154.9 (=C-O), 152.8 (C = O), 150.9 (C = N), 149.9 (=C-N), 129.8 (Ph-C), 129.4 (Ph-C), 126.7 (Ph-C), 126.6 (Ph-C), 122.7 (Ph-C), 121.3 (Ph-C), 121.2 (Ph-C), 112.3 (Ph-C), 44.4 (NCH_2_), 34.5 (NCH_3_), 13.5 (CH_3_). HR-ESI-MS: Calcd. for C_13_H_14_N_2_O_2_ [M + H]^+^: 231.1089, found: 231.1133.

#### Quinolin-4-yl morpholine-4-carboxylate (3d)

The starting material 4-hydroxyquinoline **1a** was treated with 4-morpholinecarbonyl chloride **2d** to get target derivative **3d**. Light yellow oil, yield 46% (119 mg) and 97.6% HPLC purity. ^1^H NMR 8.80 (d, *J*** **=** **4.8 Hz, 1H, Ar-H), 8.04 (d, *J*** **=** **8.4 Hz, 1H, Ar-H), 7.86 (d, *J*** **=** **8.4 Hz, 1H, Ar-H), 7.64 (t, *J*** **=** **8.0 Hz, 1H, Ar-H), 7.47 (t, *J*** **=** **8.0 Hz, 1H, Ar-H), 7.27 (d, *J*** **=** **5.2 Hz, 1H, Ar-H), 3.73–3.71 (m, 6H, 2 × OCH_2_ + NCH_2_), 3.54–3.53 (m, 2H, NCH_2_). HR-ESI-MS: Calcd. for C_14_H_14_N_2_O_3_ [M + H]^+^: 259.1038, found: 259.1089.

#### Quinolin-5-yl dimethylcarbamate (3e)

The starting material 5-hydroxyquinoline **1b** was treated with dimethylcarbamoyl chloride **2a** to get target derivative **3e**. Light yellow oil, yield 45% (97 mg) and 97.3% HPLC purity. ^1^H NMR (400 MHz, CDCl_3_) δ 8.93 (d, *J* = 2.8 Hz, 1H, Ar-H), 8.27 (d, *J*** **=** **8.4 Hz, 1H, Ar-H), 7.99 (d, *J*** **=** **8.4 Hz, 1H, Ar-H), 7.70 (t, *J*** **=** **8.4 Hz, 1H, Ar-H), 7.42 (q, *J*** **=** **4.4 Hz, 1H, Ar-H), 7.36 (d, *J*** **=** **7.6 Hz, 1H, Ar-H), 3.26 (s, 3H, NCH_3_), 3.08 (s, 3H, NCH_3_). ^13^C NMR (100 MHz, CDCl_3_) δ 154.6 (=C-O), 150.7 (C = O), 148.9 (C = N), 146.8 (=C-N), 130.1 (Ph-C), 128.9 (Ph-C), 126.8 (Ph-C), 122.8 (Ph-C), 121.2 (Ph-C), 118.7 (Ph-C), 37.0 (NCH_3_), 36.7 (NCH_3_). HR-ESI-MS: Calcd. for C_12_H_12_N_2_O_2_ [M + H]^+^: 217.0932, found: 217.0985.

#### Quinolin-5-yl diethylcarbamate (3f)

The starting material 5-hydroxyquinoline **1b** was treated with diethylcarbamoyl chloride **2b** to get target derivative **3f**. Light yellow oil, yield 43% (105 mg) and 96.7% HPLC purity. ^1^H NMR 8.92 (d, *J*** **=** **4.4 Hz, 1H, Ar-H), 8.26 (d, *J*** **=** **8.4 Hz, 1H, Ar-H), 7.99 (d, *J*** **=** **8.4 Hz, 1H, Ar-H), 7.70 (t, *J*** **=** **8.0 Hz, 1H, Ar-H), 7.42 (q, *J*** **=** **4.0 Hz, 1H, Ar-H), 7.37 (d, *J* = 8.0 Hz, 1H, Ar-H), 3.60 (q, *J*** **=** **6.8 Hz, 2H, NCH_2_), 3.44 (q, *J*** **=** **7.2 Hz, 2H, NCH_2_), 1.37 (t, *J*** **=** **6.8 Hz, 3H, CH_3_), 1.25 (t, *J*** **=** **6.8 Hz, 3H, CH_3_). HR-ESI-MS: Calcd. for C_14_H_16_N_2_O_2_ [M + H]^+^: 245.1245, found: 245.1299.

#### Quinolin-5-yl ethyl(methyl)carbamate (3g)

The starting material 5-hydroxyquinoline **1b** was treated with *N*-ethyl-*N*-methylcarbamoyl chloride **2c** to get target derivative **3g**. Light yellow oil, yield 41% (94 mg) and 96.9% HPLC purity. ^1^H NMR (400 MHz, CDCl_3_) δ 8.86 (d, *J*** **=** **3.2 Hz, 1H, Ar-H), 8.20 (d, *J*** **=** **8.4 Hz, 1H, Ar-H), 7.92 (d, *J*** **=** **8.4 Hz, 1H, Ar-H), 7.64 (t, *J*** **=** **8.4 Hz, 1H, Ar-H), 7.36 (q, *J*** **=** **4.4 Hz, 1H, Ar-H), 7.29(t, *J*** **=** **7.6 Hz, 1H, Ar-H), 3.57 + 3.40 (t, *J*** **=** **7.2 Hz, 2H, NCH_2_), 3.17 + 2.99 (s, 3 H, NCH_3_), 1.30 + 1.18 (t, *J*** **=** **6.8 Hz, 3H, CH_3_). ^13^C NMR (100 MHz, CDCl_3_) δ 154.6 (=C-O), 150.7 (C = O), 148.7 (C = N), 144.7 (=C-N), 130.2 (Ph-C), 128.9 (Ph-C), 126.9 (Ph-C), 126.8 (Ph-C), 122.9 (Ph-C), 121.2 (Ph-C), 118.7 (Ph-C), 44.4 (NCH_2_), 34.0 (NCH_3_), 13.5 (CH_3_). HR-ESI-MS: Calcd. for C_13_H_14_N_2_O_2_ [M + H]^+^: 231.1089, found: 231.1144.

#### Quinolin-5-yl morpholine-4-carboxylate (3h)

The starting material 5-hydroxyquinoline **1b** was treated with 4-morpholinecarbonyl chloride **2d** to get target derivative **3h**. Light yellow oil, yield 46% (119 mg) and 96.4% HPLC purity. ^1^H NMR 8.94 (d, *J*** **=** **4.4 Hz, 1H, Ar-H), 8.23 (d, *J*** **=** **8.4 Hz, 1H, Ar-H), 8.02 (d, *J*** **=** **8.8 Hz, 1H, Ar-H), 7.71 (t, *J*** **=** **8.0 Hz, 1H, Ar-H), 7.45 (q, *J* = 4.4 Hz, 1H, Ar-H), 7.37 (t, *J*** **=** **8.0 Hz, 1H, Ar-H), 3.85–3.83 (m, 4H, 2 × OCH_2_), 3.82–3.81 (m, 2H, NCH_2_), 3.63–3.62 (m, 2H, NCH_2_). HR-ESI-MS: Calcd. for C_14_H_14_N_2_O_3_ [M + H]^+^: 259.1038, found: 259.1086.

#### Quinolin-6-yl dimethylcarbamate (3i)

The starting material 6-hydroxyquinoline **1c** was treated with dimethylcarbamoyl chloride **2a** to get target derivative **3i**. Light yellow oil, yield 47% (102 mg) and 97.2% HPLC purity. ^1^H NMR 8.71 (d, *J*** **=** **2.0 Hz, 1H, Ar-H), 8.44 (s, 1H, Ar-H), 7.99 (d, *J*** **=** **8.4 Hz, 1H, Ar-H), 7.72 (d, *J*** **=** **8.4 Hz, 1H, Ar-H), 7.59 (t, *J* = 8.0 Hz, 1H, Ar-H), 7.49 (t, *J*** **=** **8.0 Hz, 1H, Ar-H), 7.16 (s, 1H, Ar-H), 3.73 (s, 3H, NCH_3_), 3.54 (s, 3H, NCH_3_). HR-ESI-MS: Calcd. for C_12_H_12_N_2_O_2_ [M + H]^+^: 217.0899, found: 217.0956.

#### Quinolin-6-yl diethylcarbamate (3j)

The starting material 6-hydroxyquinoline **1c** was treated with diethylcarbamoyl chloride **2b** to get target derivative **3j**. Light yellow oil, yield 44% (107 mg) and 97.0% HPLC purity. ^1^H NMR 8.76 (d, *J*** **=** **2.0 Hz, 1H, Ar-H), 8.49 (s, 1H, Ar-H), 7.98 (d, *J*** **=** **7.6 Hz, 1H, Ar-H), 7.69 (d, *J*** **=** **8.0 Hz, 1H, Ar-H), 7.55 (t, *J* = 7.6 Hz, 1H, Ar-H), 7.45 (t, *J*** **=** **8.0 Hz, 1H, Ar-H), 3.60–3.58 (m, 2H, NCH_2_), 3.47–3.45 (m, 2H, NCH_2_), 1.35 (t, *J*** **=** **6.8 Hz, 3H, CH_3_), 1.27 (t, *J*** **=** **7.2 Hz, 3H, CH_3_). HR-ESI-MS: Calcd. for C_14_H_16_N_2_O_2_ [M + H]^+^: 245.1245, found: 245.1293.

#### Quinolin-6-yl ethyl(methyl)carbamate (3k)

The starting material 6-hydroxyquinoline **1c** was treated with *N*-ethyl-*N*-methylcarbamoyl chloride **2c** to get target derivative **3k**. Light yellow oil, yield 38% (87 mg) and 97.1% HPLC purity. ^1^H NMR 8.75 (d, *J*** **=** **2.0 Hz, 1H, Ar-H), 8.49 (s, 1H, Ar-H), 7.97 (d, *J*** **=** **8.0 Hz, 1H, Ar-H), 7.68 (d, *J*** **=** **8.0 Hz, 1H, Ar-H), 7.55 (t, *J* = 8.0 Hz, 1H, Ar-H), 7.45 (t, *J*** **=** **8.0 Hz, 1H, Ar-H), 3.43 (q, *J*** **=** **7.2 Hz, 2H, NCH_2_), 3.02 (s, 3H, NCH_3_), 1.18 (t, *J*** **=** **6.8 Hz, 3H, CH_3_). HR-ESI-MS: Calcd. for C_13_H_14_N_2_O_2_ [M + H]^+^: 231.1089, found: 231.1126.

#### Quinolin-6-yl morpholine-4-carboxylate (3l)

The starting material 6-hydroxyquinoline **1c** was treated with 4-morpholinecarbonyl chloride **2d** to get target derivative **3l**. Light yellow oil, yield 48% (124 mg) and 96.7% HPLC purity. ^1^H NMR 8.77 (d, *J*** **=** **2.0 Hz, 1H, Ar-H), 8.50 (s, 1H, Ar-H), 7.97 (d, *J*** **=** **7.6 Hz, 1H, Ar-H), 7.68 (d, *J*** **=** **8.0 Hz, 1H, Ar-H), 7.54 (t, *J* = 7.6 Hz, 1H, Ar-H), 7.44 (t, *J*** **=** **8.0 Hz, 1H, Ar-H), 3.78–3.71 (m, 6H, 2 × OCH_2_ + NCH_2_), 3.63–3.57 (m, 2H, NCH_2_). HR-ESI-MS: Calcd. for C_14_H_14_N_2_O_3_ [M + H]^+^: 259.1038, found: 259.1077.

#### Quinolin-8-yl dimethylcarbamate (3m)

The starting material 8-hydroxyquinoline **1d** was treated with dimethylcarbamoyl chloride **2a** to get target derivative **3m**. Light yellow oil, yield 51% (110 mg) and 97.5% HPLC purity. ^1^H NMR (400 MHz, CDCl_3_) δ 8.91 (d, *J* = 2.4 Hz, 1H, Ar-H), 8.11 (d, *J* = 6.8 Hz, 1H, Ar-H), 7.65 (d, *J* = 6.0 Hz, 1H, Ar-H), 7.51–7.44 (m, 2H, 2 × Ar-H), 7.37 (q, *J*** **=** **4.0 Hz, 1H, Ar-H), 3.26 (s, 3H, NCH_3_), 3.05 (s, 3H, NCH_3_). HR-ESI-MS: Calcd. for C_12_H_12_N_2_O_2_ [M + H]^+^: 217.0932, found: 217.0984.

#### Quinolin-8-yl diethylcarbamate (3n)

The starting material 8-hydroxyquinoline **1d** was treated with diethylcarbamoyl chloride **2b** to get target derivative **3n**. Light yellow oil, yield 46% (112 mg) and 98.1% HPLC purity. ^1^H NMR (400 MHz, CDCl_3_) δ 8.90 (d, *J*** **=** **2.8 Hz, 1H, Ar-H), 8.14 (d, *J*** **=** **7.2 Hz, 1H, Ar-H), 7.67 (d, *J*** **=** **6.4 Hz, 1H, Ar-H), 7.53–7.46 (m, 2H, 2 × Ar-H), 7.39 (q, *J*** **=** **4.0 Hz, 1H, Ar-H), 3.64 (q, *J*** **=** **6.8 Hz, 2H, NCH_2_), 3.45 (q, *J*** **=** **6.8 Hz, 2H, NCH_2_), 1.40 (t, *J*** **=** **6.8 Hz, 3H, CH_3_), 1.25 (t, *J*** **=** **6.8 Hz, 3H, CH_3_). HR-ESI-MS: Calcd. for C_14_H_16_N_2_O_2_ [M + H]^+^: 245.1245, found: 245.1296.

#### Quinolin-8-yl ethyl(methyl)carbamate (3o)

The starting material 8-hydroxyquinoline **1d** was treated with *N*-ethyl-*N*-methylcarbamoyl chloride **2c** to get target derivative **3o**. Light yellow oil, yield 42% (97 mg) and 98.1% HPLC purity. ^1^H NMR (400 MHz, CDCl_3_) δ 8.91 (d, *J* = 2.8 Hz, 1H, Ar-H), 8.13 (d, *J* = 6.8 Hz, 1H, Ar-H), 7.67 (d, *J* = 7.2 Hz, 1H, Ar-H), 7.53–7.46 (m, 2H, 2 × Ar-H), 7.38 (q, *J*** **=** **4.0 Hz, 1H, Ar-H), 3.67 + 3.47 (q, *J*** **=** **7.2 Hz, 2H, NCH_2_), 3.26 + 3.05 (s, 3H, NCH_3_), 1.38 + 1.24 (t, *J*** **=** **7.2 Hz, 3H, CH_3_). HR-ESI-MS: Calcd. for C_13_H_14_N_2_O_2_ [M + H]^+^: 231.1089, found: 231.1136.

#### Quinolin-8-yl morpholine-4-carboxylate (3p)

The starting material 8-hydroxyquinoline **1d** was treated with 4-morpholinecarbonyl chloride **2d** to get target derivative **3p**. Light yellow oil, yield 38% (98 mg) and 97.2% HPLC purity. ^1^H NMR (400 MHz, CDCl_3_) δ 8.91 (d, *J*** **=** **8.0 Hz, 1H, Ar-H), 8.15 (d, *J*** **=** **8.4 Hz, 1H, Ar-H), 7.69 (d, *J* = 7.6 Hz, 1H, Ar-H), 7.52 (t, *J*** **=** **8.0 Hz, 1H, Ar-H), 7.48 (d, *J*** **=** **7.6 Hz, 1H, Ar-H), 7.40 (q, *J*** **=** **4.0 Hz, 1H, Ar-H), 3.90–3.84 (m, 4H, 2 × OCH_2_), 3.83–3.78 (m, 2H, NCH_2_), 3.64–3.62 (m, 2H, NCH_2_). HR-ESI-MS: Calcd. for C_14_H_14_N_2_O_3_ [M + H]^+^: 259.1038, found: 259.1086.

#### Quinolin-8-yl diisopropylcarbamate (3q)

The starting material 8-hydroxyquinoline **1d** was treated with diisopropylcarbamoyl chloride **2e** to get target derivative **3q**. Light yellow oil, yield 40% (109 mg) and 98.1% HPLC purity. ^1^H NMR (400 MHz, CDCl_3_) δ 8.86 (d, *J* = 3.2 Hz, 1H, Ar-H), 8.08 (d, *J*** **=** **8.0 Hz, 1H, Ar-H), 7.61 (t, *J* = 4.0 Hz, 1H, Ar-H), 7.48–7.46 (m, 2H, 2 × Ar-H), 7.34 (q, *J*** **=** **4.0 Hz, 1H, Ar-H), 4.26–4.24 (m, 1H, NCH), 4.05–4.04 (m, 1H, NCH), 1.44 (s, 6H, 2 × CH_3_), 1.34 (s, 6H, 2 × CH_3_). HR-ESI-MS: Calcd. for C_16_H_20_N_2_O_2_ [M + H]^+^: 273.1558, found: 273.1606.

### Biological activity

#### Inhibition experiments of AChE and BuChE

Acetylcholinesterase (*ee*AChE, from the electric eel; hAChE, from human erythrocytes AChE), butyrylcholinesterase (eqBuChE, from equine serum; hBuChE, human serum BuChE), 5,5′-dithiobis-2-nitrobenzoic acid (Ellman’s reagent, DTNB), acetylthiocholine chloride (ATC), and butyrylthiocholine chloride (BTC) were purchased form Sigma Aldrich. The determination of AChE and BuChE inhibitory potency of the synthesised compounds were evaluated by Ellman assay, the detailed procedure referred to our previous work^26^^,^^27^.

#### Anti-inflammatory property

BV-2 cells were cultured in DMEM containing 10% foetal bovine serum, 100 U/mL penicillin, 100 U/mL streptomycin, and 1% nonessential amino acid at 37 °C with 5% CO_2_.

BV-2 cells in logarithmic growth stage were digested and centrifuged, and then resuspended in DMEM medium containing 1% penicillin-streptomycin and 10% foetal bovine serum. The cells was adjusted to 1.2 × 10^4^ cells/well, and then seeded evenly in 96-well cell culture plates. The cells were incubated for 36 h at 37 °C with 5% CO_2_. After incubation, the cells were added to different concentrations (10 and 2 µM) of tested compounds, and incubated for 24 h at 37 °C with 5% CO_2_. Then, 10 µL CCK-8 solution was added to each well, and incubated for another 1 h at 37 °C with 5% CO_2_. The OD values were tested at 450 nM. The cell viability (%) = {(OD_drug_−OD_blank_)}/{(OD_control_−OD_blank_)} × 100%.

BV-2 cells (2 × 10^5^ cells/well) were seeded in a 24-well cell culture plates. The cells were divided into blank control group, model group (1 µg/mL LPS), LPS + 2 µM compound group, LPS + 1 µM compound group. Each drug group was pre-treated with corresponding concentration of drug solution for 8 h. After 8 h, the culture medium was aspirated out. Except for the blank control group, which was added with DMEM medium, the other groups were stimulated with 1 µg/mL LPS for 24 h. Then cell supernatant was aspirated and centrifuged at 3000 rpm for 3 min. The content of NO in cell culture supernatant was determined by Griess method, and the levels of cytokines IL-6 and IL-1*β* in cell culture supernatant were determined by IL-6 ELISA kit and IL-1*β* ELISA kit, respectively.

#### Neuroprotective effect on Aβ_25-35_-induced PC12 cells injury

1. Preparation of A*β*_25-35_ aggregation. Aβ_25-35_ was purchased from sigma. 1 mg Aβ_25-35_ was dissolved in 943 µL of sterilised deionised water to obtain 1 mM stock solution, aliquoted, –20 °C preservation. Removing the appropriate volume in a 48-well plate and incubated in a 37 °C incubator for two days to allow its ageing to accumulate and produce neurotoxicity. After aggregation was completed, the solution was diluted with PBS to get 25 µM solution of A*β*_25-35_ aggregation.
2. Neuroprotective effects of compounds on A*β*_25-35_-induced PC12 cells injury

The PC12 cells during the logarithmic growth phase were digested and centrifuged with pancreatic enzyme solution, the cells were resuspended by adding the prepared media solution, and piped evenly with a pipette to seed in a 96-well cell culture plate (cell concentration adjusted to 2.0 × 10^4^ cell·mL^−1^). Each well was added 100 µL complete medium was added to each well and incubated at 37 °C and 5% CO_2_ for 24 h.

After incubation for 24 h, PC12 cells were divided into blank group, control group, A*β*_25-35_ model group and tested compounds group (5, 10, 20 µM). Then the original medium was discarded, and 100 µL new complete medium were added to PC12 cells, and the tested compounds group was added to 100 µL complete medium containing different concentrations (5, 10, and 20 µM) of tested compounds, incubated for 2 h at 37 °C, 5% CO_2_ conditions. After incubation for 2 h, 10 µL A*β*_25-35_ solution was added to the tested compounds groups and model group (the final concentration of A*β*_25-35_ was 25 µM), and incubated for 24 h at 37 °C, 5% CO_2_ conditions, 10 µL CCK-8 was added to each well and further incubated for 1 h at 37 °C and 5% CO_2_. Finally, the OD value was measured at 450 nm using a microplate reader, and each concentration set three wells. The cell survival rate (%) was determined according to the following formula: cell viability (%) = {(OD_drug_−OD_blank_)}/{(OD_control_−OD_blank_)}.

#### The stability studies

1. The artificial gastrointestinal fluids were prepared on the basis of the standard method described in China Pharmacopoeia (version 2015). The artificial gastric fluid consisted of HCl (0.045 mol/L) and pepsin (10 g/L), while the artificial intestinal fluid consisted of trypsin (10 g/L) and KH_2_PO_4_ (6.8 g/L), and the pH was adjusted to 6.8 with 0.1 mol/L NaOH. The blank gastric fluid and blank intestinal fluid was similar as described above without pepsin and trypsin. Compound **3f** (1 mg/mL, n = 3) was incubated with artificial gastric fluid、blank gastric fluid 、artificial intestinal fluid and blank intestinal fluid at 37 ◦C, and 600 µL methanol was then added into the reagent to stop reaction at time of 0, 0.5, 1, 2, 3, 4, and 8 h. Supernatant was collected by centrifugation at 13 000 rpm for 10 min and analysed by UPLC-MS/MS. The data were processed by GraphPad Prism 8.0 software.

UPLC-MS/MS method: Targeted analytes were separated using a Agilent xbridge aq C18 column (4.6 mm × 250 mm, 5 µm, USA) at a flow rate of 0.4 ml/min. The mobile phase consisted of solvent A (deionised water with 0.1% formic acid) and solvent B (acetonitrile with 0.1% formic acid). The gradient elution program was as follows: 0–0.5 min (10% B); 0.5–2.5 min (10–98% B); 2.5–3.5 min (98% B); 3.5–3.6 min (98–5% B); 3.6–5.0 min (10% B). The autosampler temperature was maintained at 12 °C and the injection volume was set at 1 µL.

The mass spectrometer was operated in positive ion mode through multiple-reaction monitoring. The parameters of the mass spectrometer were as follows: capillary ionisation voltage of 3500 V: source gas temperature of 300 °C gas flow of 11 L/min. The detailed optimised MS/MS parameters are presented in Table 3.

**Table 3.** Mass spectrometry parameters for **3f**.

Analyte	Precursor ion	Production ion	Fragmentor	Collision energy (eV)
**3f**	217.1	160	100	16
		72.1	100	24

2.The stability of **3f** was performed in rat liver microsomes. The rat liver microsomes was purchased from Dalian Meilun Biotechnology Co., Ltd, DaLian, China. Briefly, solution A (10 mL) contained G-6-P-Na_2_ (200 mg), NADPNa_2_ (200 mg), MgCl_2_ (133 mg), and H_2_O was used as a solvent. Solution B (25 mL) contained G-6-P-DH (1000 U), Na-Citrate2 (200 mg), and H_2_O was used as a solvent. NADPH solution (1 mM) was composed by solution A and solution B (*v/v* = 5:1). Compound **3f** was dissolved in DMSO to obtain 1 mg/mL stock solution. Rat liver microsomes was added to **3f** solution (the final concentration of **3f** and rat liver microsomes was 10 µg/mL and 0.5 mg/mL, respectively) (n = 3). The reaction was started by the addition of NADPH. The total mixture (200 µL) was incubated at 37 °C. Cold methanol (600 µL) was added at 0, 5, 15, 20, 30, 45 and 60 min, respectively, to stop the reaction. Samples were centrifuged at 13000 rpm for 10 min at 4 °C, and 100 µL supernatant was removed and analysed by UPLC-MS/MS. The stability results were presented as % remaining *vs.* time. The experiments were conducted in triplicate.
3.The stability of **3f** was performed in rat plasma. Briefly, compounds **3f** (1 mg/mL) were dissolved in DMSO, and the reaction (10 µg/mL, n = 3) was prepared by mixing 198 µL plasma with 2 µL compounds solution, then incubated in 37 ◦C and stopped by adding 600 µL methanol at time of 0, 0.5, 1, 1.5, 2 and 3 h, followed by centrifugation at 13 000 rpm for 10 min at 4 °C. The supernatant was dried and redissolved in 100 µL methanol. After centrifugation, the supernatant was analysed by UPLC-MS/MS. The stability data were presented as remaining percentage vs time.

#### Effect of 3f on AlCl_3_-induced zebrafish AD

1. Effect of **3f** on the survival rate of zebrafish embryos. Zebrafish embryos (4 hpf) at 4h post-fertilisation were selected and treated with seven concentrations (0.65, 1.3, 2.6, 5.2, 10.3, 20.5 and 40.9 µM) of **3f**, and cultured at 28 °C. The survival rate of zebrafish embryos was observed and recorded every 24h, and observation was stopped when zebrafish embryos developed to 120 hpf. Each experiments were performed by three independent groups (n = 10).
2. Effect of **3f** on zebrafish morphology. Zebrafish embryos (4 hpf) were selected and treated with six concentrations (0.65, 1.3, 2.6, 5.2, 10.3, and 20.5 µM) of **3f**, and incubated at 28 °C. When the culture was to be 120hpf, photographic observations were made under a body microscope (RM2235; Leica). And the body length, pericardial oedema, swim bladder area, liver area, and liver fluorescence intensity of zebrafish were measured by ImageJ software.
3. Zebrafish experimental grouping. Control group: zebrafish were cultured at 4-168 hpf of development and placed in ZR solution. Model group: zebrafish embryos at 4hpf were selected and treated with 140 µM AlCl_3_ until to 72 hpf, and the model was established when they reached 120 hpf. When the zebrafish embryos developed to 120 hpf, 8 µM of DPZ and **3f** (0.08, 0.16 and 0.32 µM) were added for 24h. When the zebrafish embryos developed to 168 hpf, the experiments were completed and performed further subsequent investigations.
4. Behavioural assay of zebrafish. The zebrafish embryos’ swimming activity (swimming distance, swimming speed, and reactivity) under alternating light and dark conditions were measured using a behavioural system (Zebrafish3.3; ViewPoint). At 168 hpf, zebrafish were placed in 24-well plates (28°C), and after 10 min of acclimatisation under light conditions, swimming activity under light conditions (10 min), and swimming activity under dark conditions (10 min) were examined, alternating three times. All treatment groups had 3 replicates with 12 embryos each.
5. Detection of ACh content and AChE activity. Zebrafish embryos at 168 hpf were collected (60 zebrafish per group, 3 replicates per group), the collected zebrafish were washed and homogenised with 10x PBS (pH = 7.2), then centrifuged at 8000g for 10min to obtain the supernatant. Assays were performed according to the manufacturer’s protocol (ACh: BC2025; Solarbio, ACh: NM9191201; Meimian).
6. Statistical analysis. All the data were expressed as mean ± SEM and statistical analysis were performed using GraphPad Prism software. The significance of differences were determined using one-way ANOVA. Data were expressed as mean ± standard deviation, and *p < 0.05 indicates a significant difference.

## Supplementary Material

Supplemental Material
