# Supplementary material for: Design, synthesis and evaluation of quinoline-O-carbamate derivatives as multifunctional agents for the treatment of Alzheimer’s disease
Source: J Enzyme Inhib Med Chem. 2023 Jan 23;38(1):2169682. doi: 10.1080/14756366.2023.2169682 (PMC9873282; doi:10.1080/14756366.2023.2169682)
Supplement: Supplemental Material [file IENZ_A_2169682_SM9827.pdf]

Supporting information

Design, synthesis and evaluation of quinoline-*O*-carbamate derivatives as multifunctional agents for the treatment of Alzheimer's disease

Hongsong Chen[1,#], Jing Mi[2,#], Sen Li[4,#], Zhengwei Liu[2], Jing Yang[2], Rui Chen[5], Yujie Wang[5], Yujuan Ban[5], Yi Zhou[2,*], Wu Dong[1,*], Zhipei Sang[*, 2, 3].

[1]Inner Mongolia Key Laboratory of Toxicant Monitoring and Toxicology, College of Animal Science and Technology. Inner Mongolia Minzu University, Tongliao, Inner Mongolia, 028000, China

[2]College of Chemistry and Pharmaceutical Engineering, Nanyang Normal University, Nanyang, Henan, 473061, China

[3]School of Pharmaceutical Sciences, Hainan University, Haikou, Hainan, 570228, China

[4]Department of Orthopaedics Surgery, Nanyang Central Hospital, Nanyang, Henan, 473009, China

[5]State Key Laboratory of Functions and Applications of Medicinal Plants, Guizhou Provincial Engineering Technology Research Center for Chemical Drug R&D, Guizhou Medical University, Guiyang, Guizhou, 550004, China

**Corresponding Author*.

E-mail: sangzhipei@126.com (Zhipei Sang)

E-mail: dongwu@imun.edu.cn (Wu Dong)

E-mail: yzhou0503@163.com (Yi Zhou)

#These authors contributed equally.

Representative spectra for the synthesized compounds

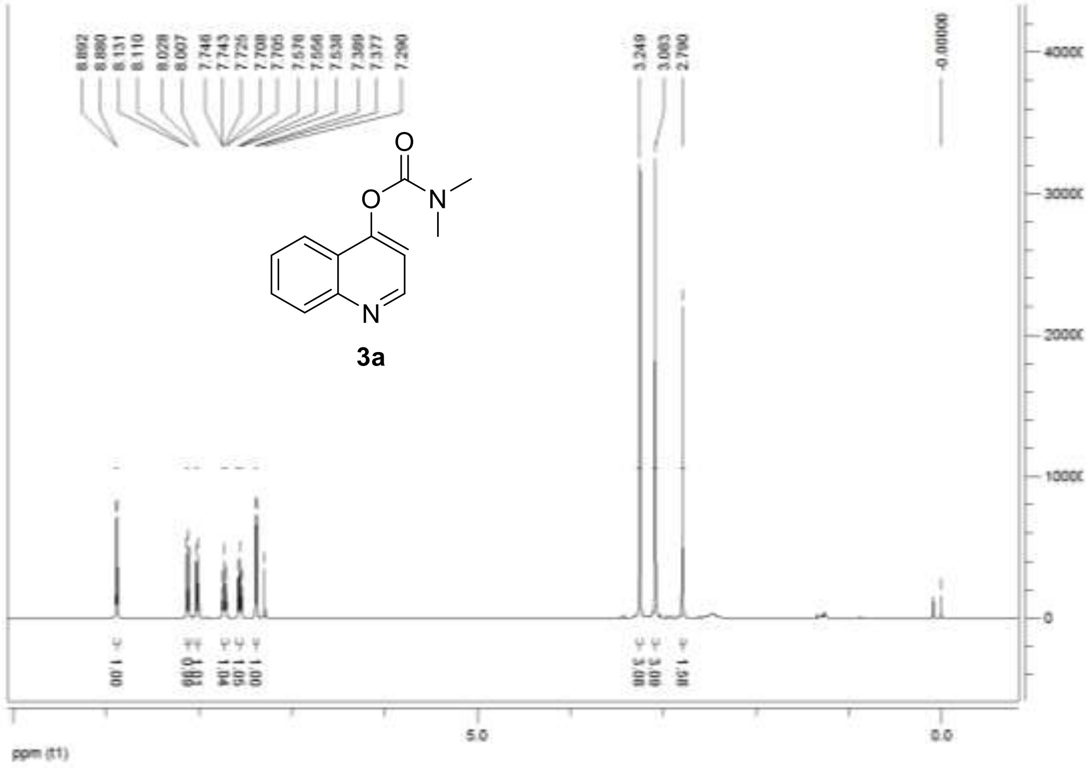

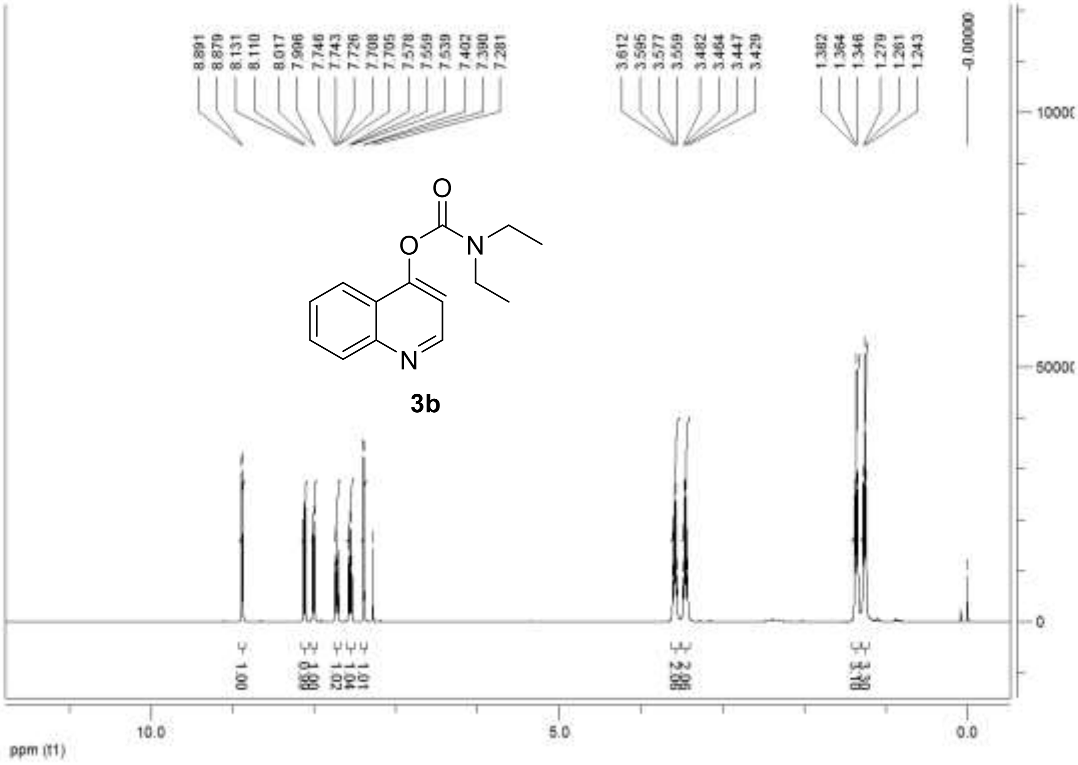

8.891
8.879
8.131
8.110
8.017
7.996
7.746
7.743
7.726
7.708
7.705
7.578
7.559
7.539
7.402
7.390
7.281
3.612
3.595
3.577
3.559
3.482
3.464
3.447
3.429
1.382
1.364
1.346
1.279
1.261
1.243
-0.00000
O
O
N
N
3b
1.00
1.04
1.01
2.08
3.10
10.0
5.0
0.0
ppm (t1)

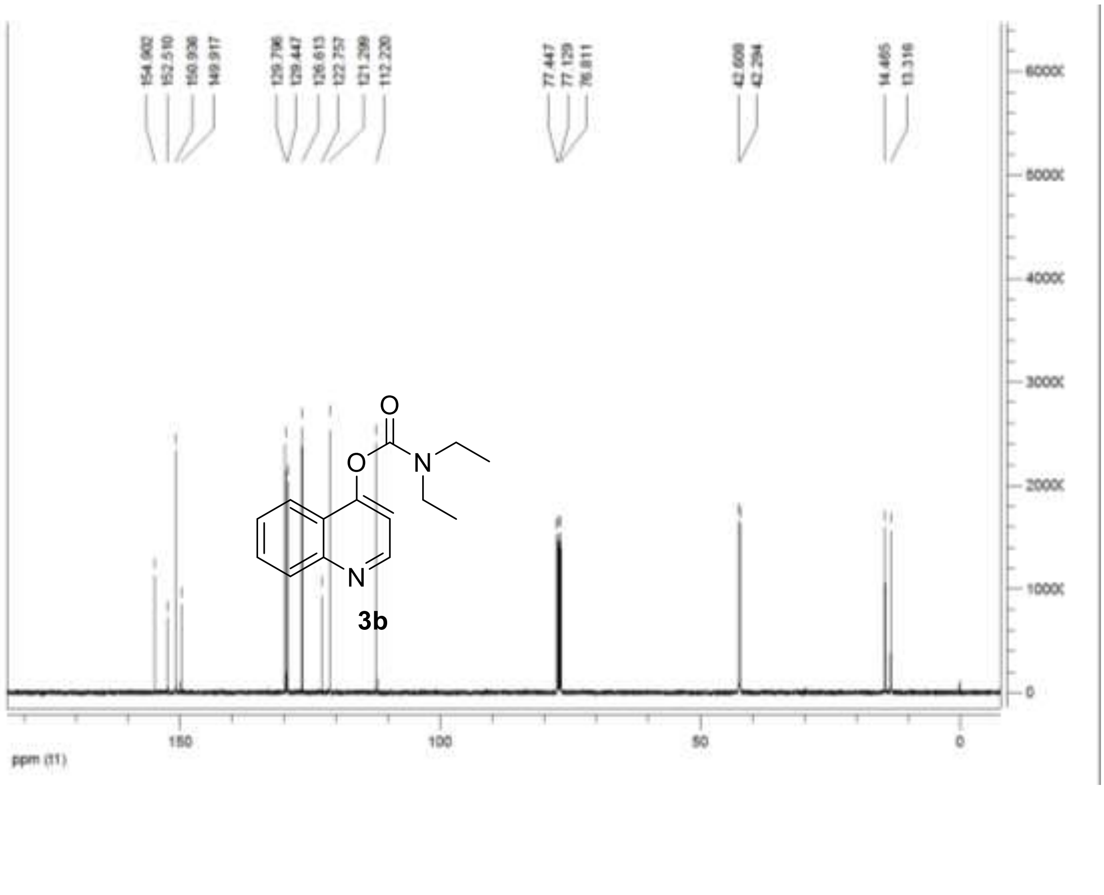

154.902
152.510
150.936
149.917
129.796
129.447
126.613
122.757
121.299
112.220
77.447
77.129
76.811
42.608
42.294
14.465
13.316
O
O
N
N
3b
150
100
50
0
ppm (t1)

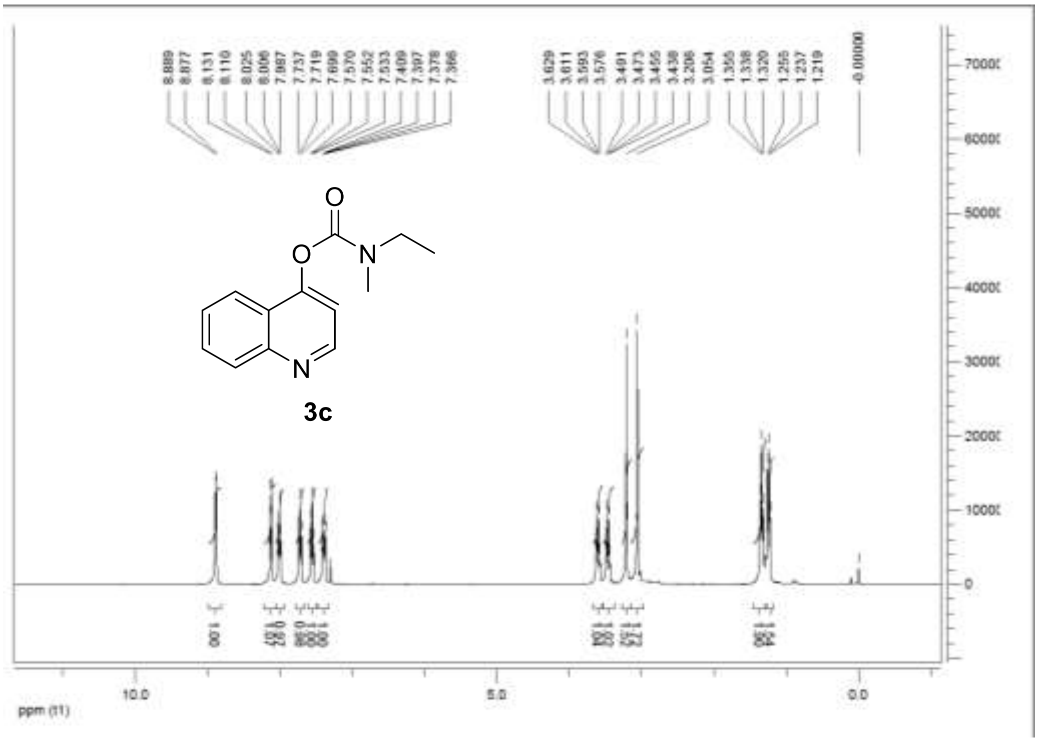

3c

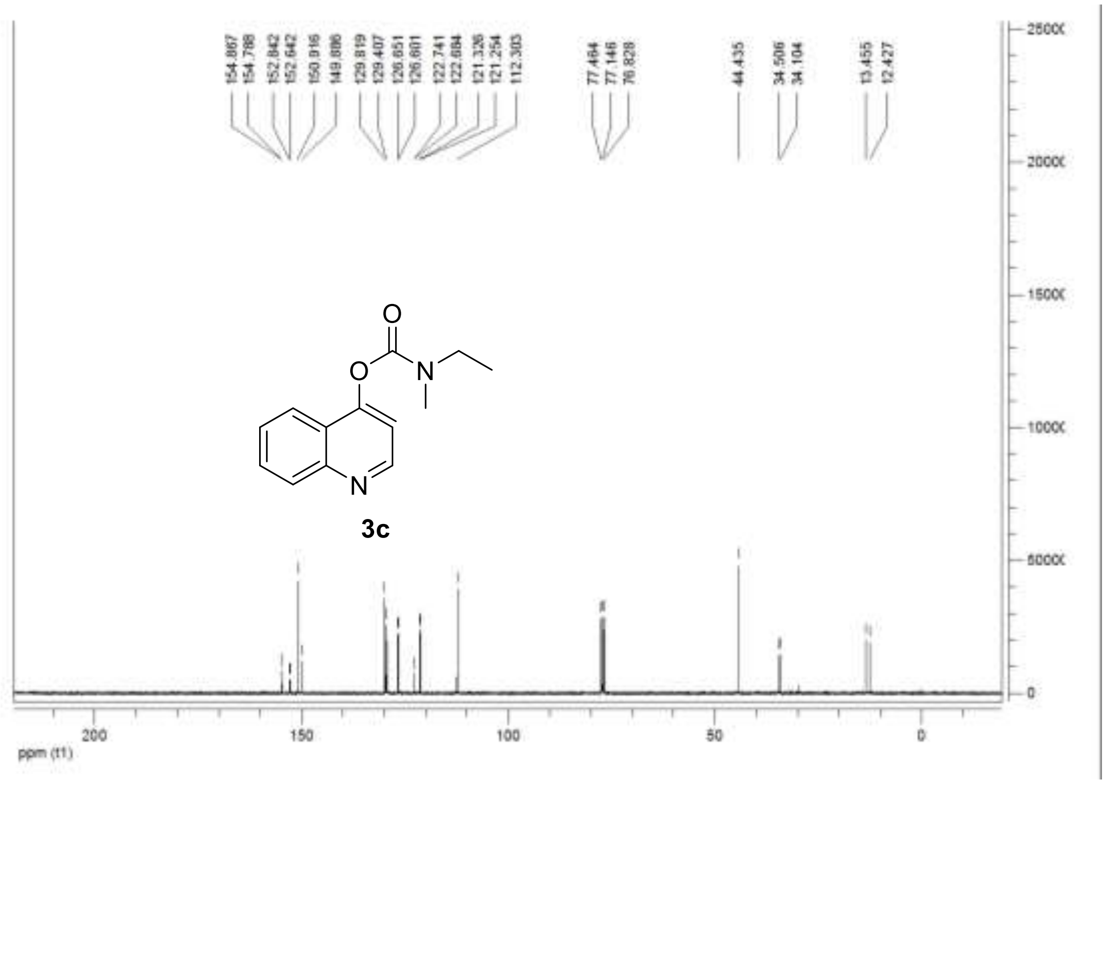

3c

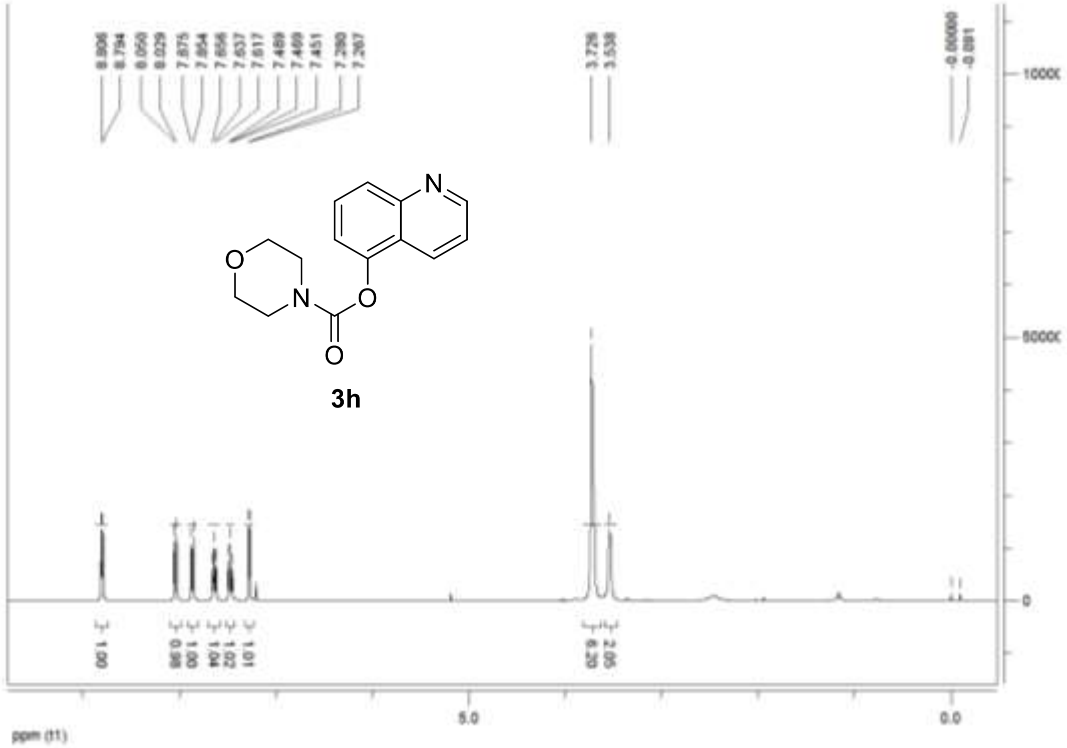

8.806
8.794
8.050
8.029
7.875
7.854
7.656
7.637
7.617
7.489
7.469
7.451
7.280
7.267
3.726
3.538
-0.00000
-0.081
3h
1.00
0.98
1.00
1.04
1.02
1.01
6.20
2.05
5.0
0.0
ppm (t1)

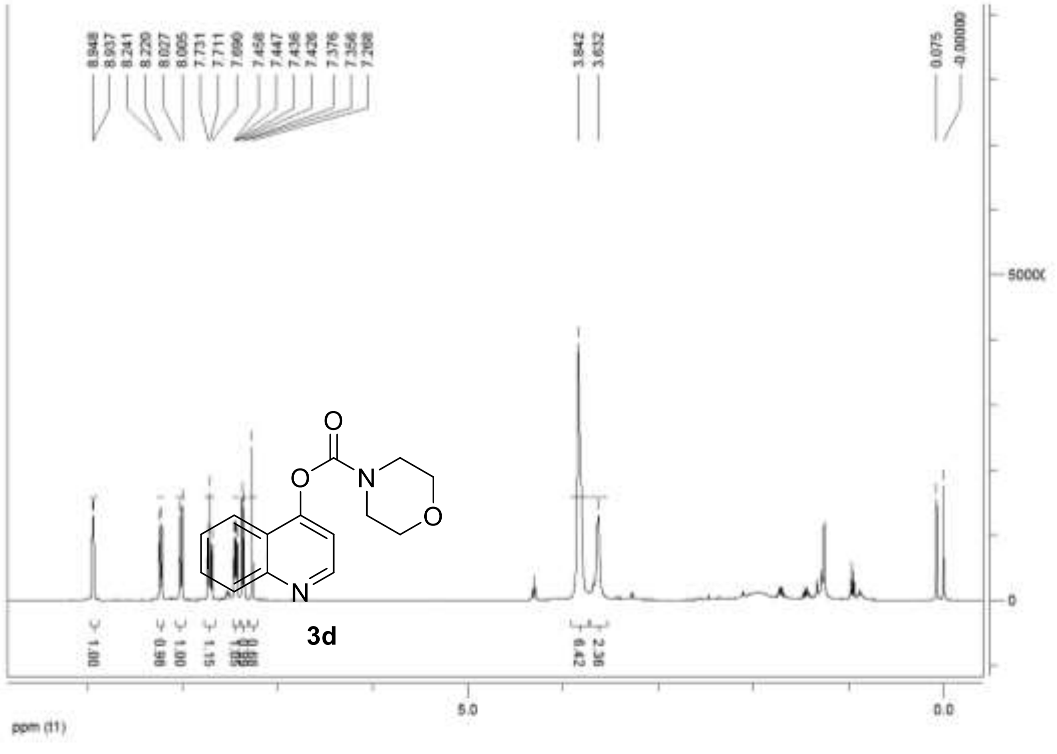

8.948
8.937
8.241
8.220
8.027
8.005
7.731
7.711
7.690
7.458
7.447
7.436
7.426
7.376
7.356
7.268
3.842
3.632
0.075
-0.00000
50000
3d
1.00
0.96
1.00
1.15
6.42
2.36
5.0
0.0
ppm (t1)

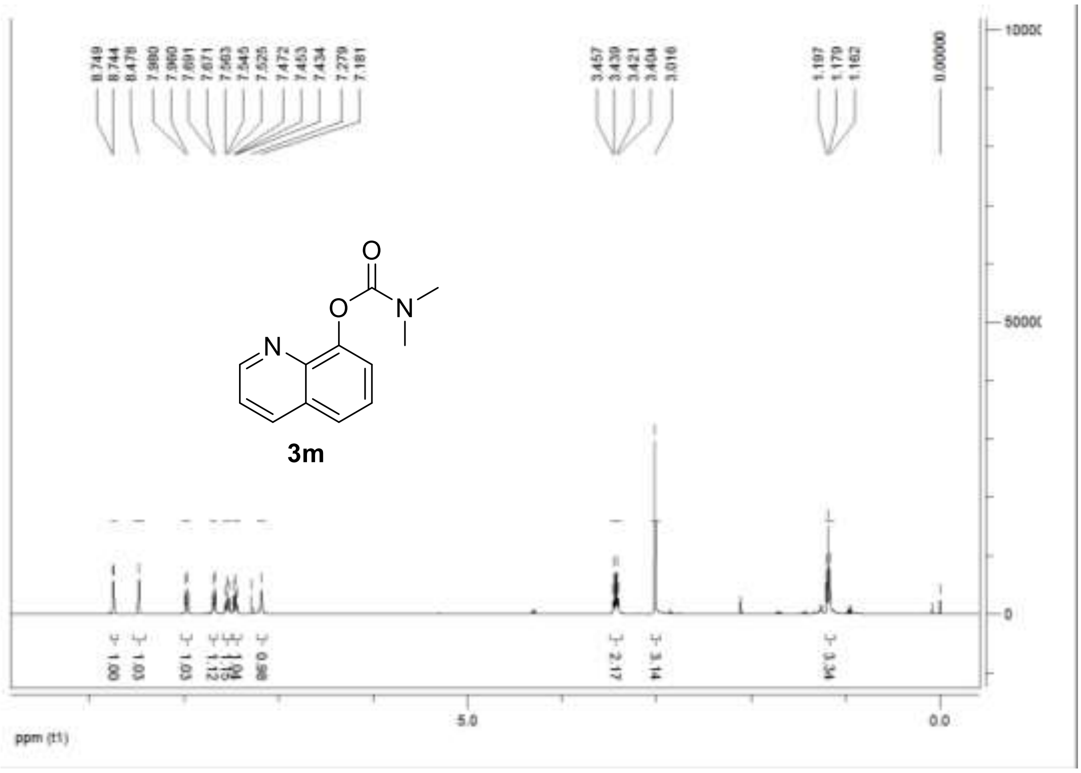
8.749
8.744
8.478
7.980
7.960
7.691
7.671
7.563
7.545
7.525
7.472
7.453
7.434
7.279
7.181
3.457
3.439
3.421
3.404
3.016
1.197
1.179
1.162
0.00000
O
O
N
N
3m
1.00
1.03
1.03
1.12
2.17
3.14
3.34
5.0
0.0
ppm (t1)
10000
50000
0

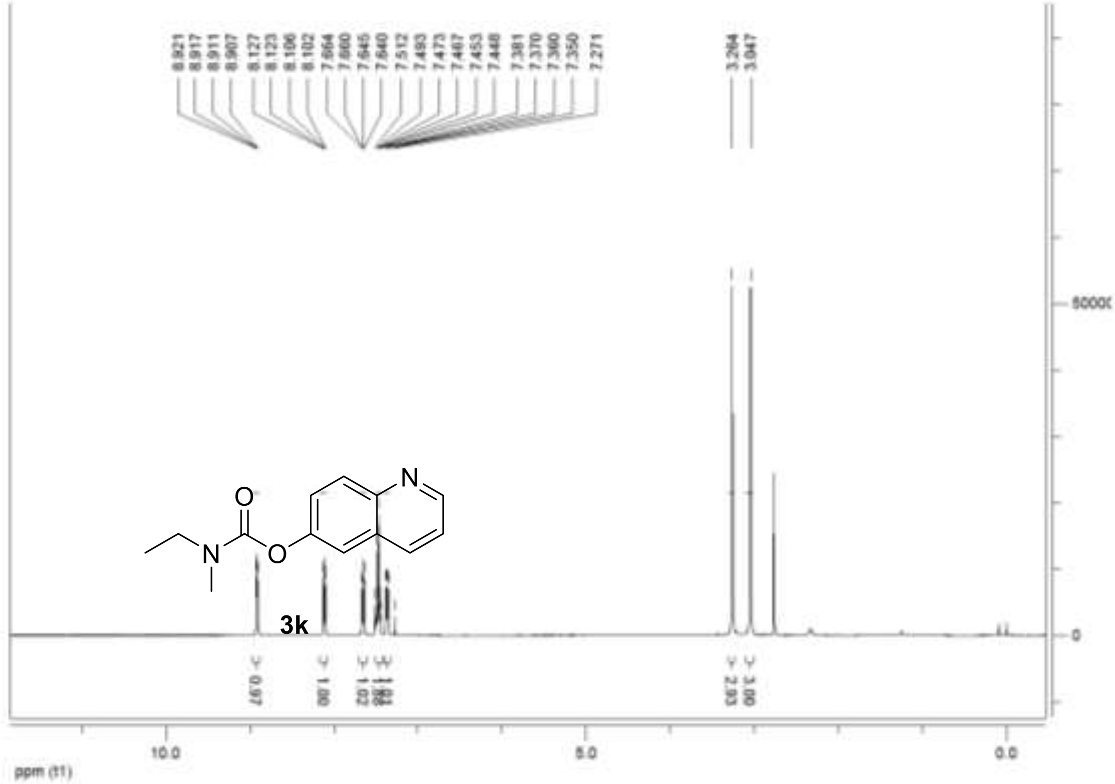
8.921
8.917
8.911
8.907
8.127
8.123
8.106
8.102
7.664
7.660
7.645
7.640
7.512
7.493
7.473
7.467
7.453
7.448
7.381
7.370
7.360
7.350
7.271
3.264
3.047
O
N
O
N
3k
0.97
1.00
1.02
2.93
3.00
10.0
5.0
0.0
ppm (t1)
50000
0

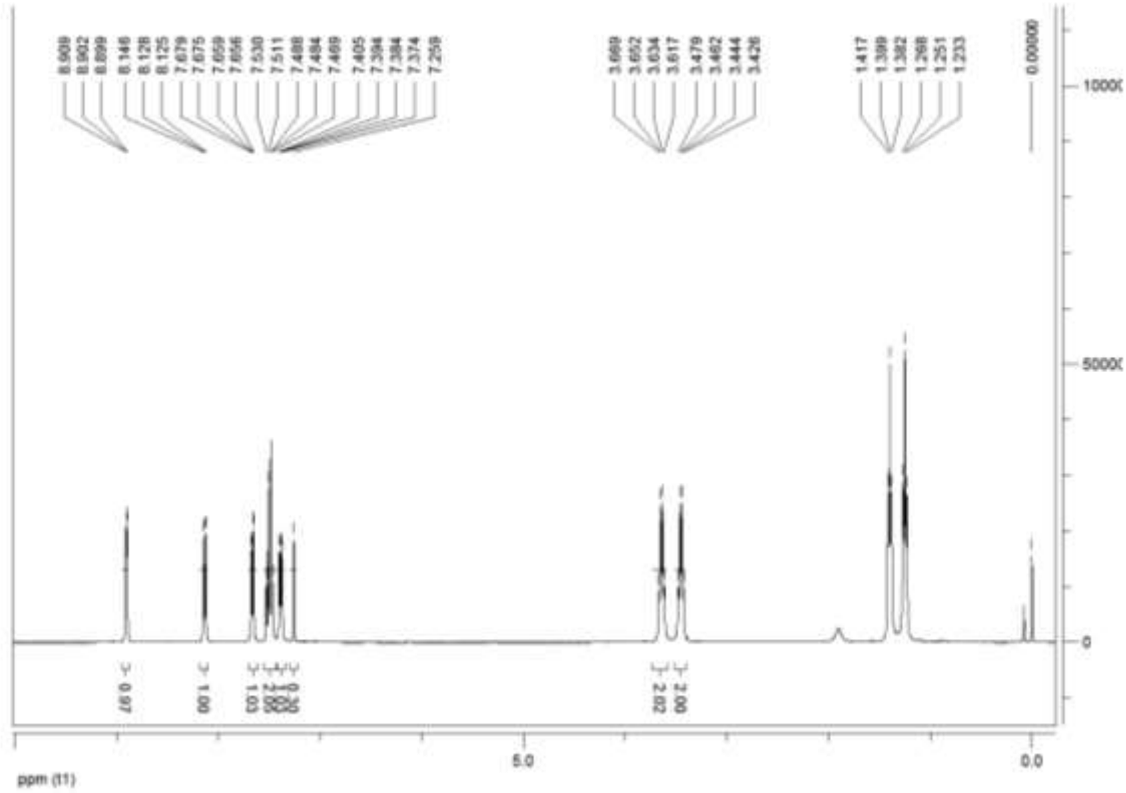
8.909
8.902
8.899
8.146
8.128
8.125
7.679
7.675
7.659
7.656
7.530
7.511
7.488
7.484
7.469
7.405
7.394
7.384
7.374
7.259
3.669
3.652
3.634
3.617
3.479
3.462
3.444
3.426
1.417
1.399
1.382
1.268
1.251
1.233
0.00000
10000
50000
0
0.97
1.00
1.03
2.05
0.90
2.02
2.00
5.0
0.0
ppm (t1)

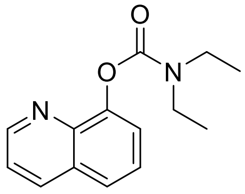
O
O
N
N

3n

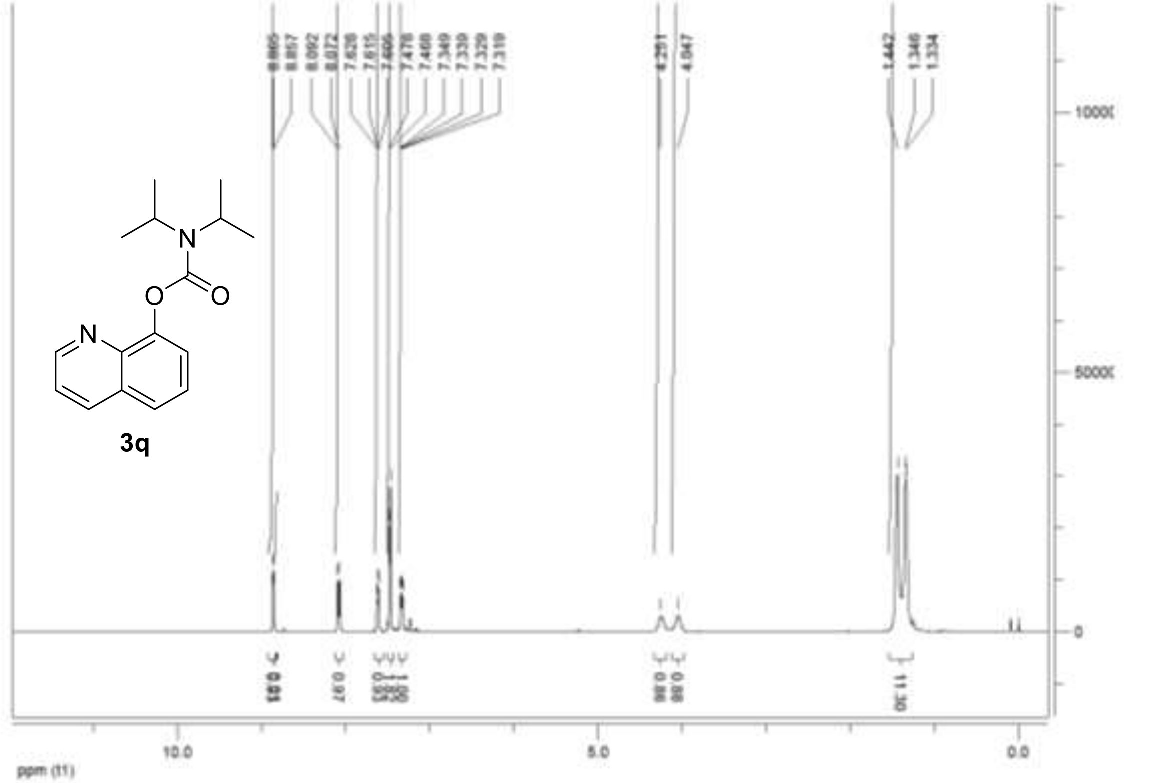

8.865
8.857
8.092
8.072
7.628
7.615
7.606
7.476
7.468
7.349
7.339
7.329
7.319
4.251
4.047
1.442
1.346
1.334
10000
50000
0
0.97
0.86
0.88
11.30
10.0
5.0
0.0
ppm (t1)

3q

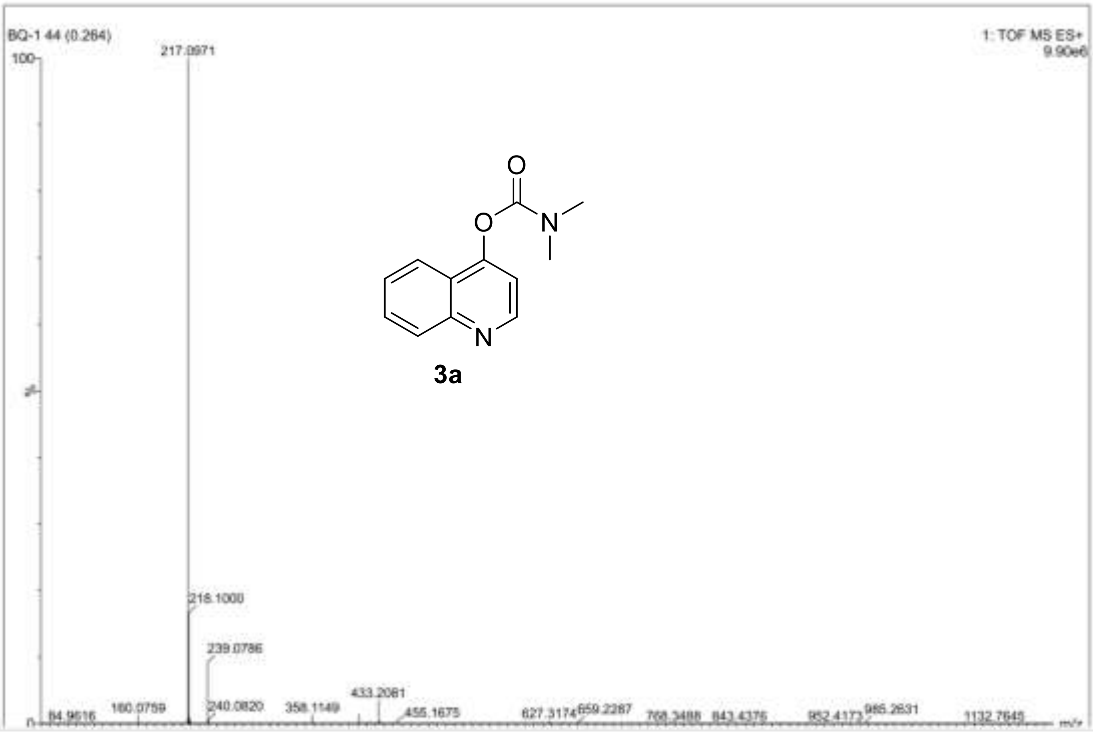

BQ-1 44 (0.264)
1: TOF MS ES+
9.90e6
100
%
0
217.0971
218.1000
239.0786
240.0820
84.9616
160.0759
358.1149
433.2061
455.1675
627.3174
659.2287
768.3488
843.4376
952.4173
985.2631
1132.7645
m/z
O
N
3a

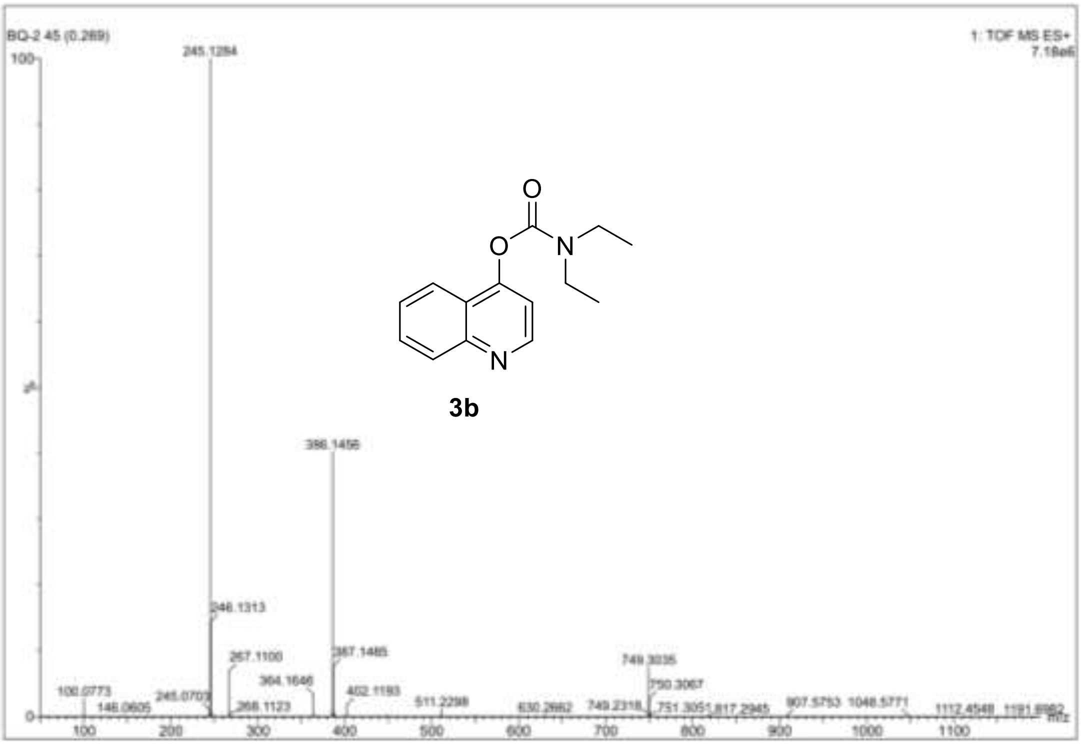

BQ-2 45 (0.269)
1: TOF MS ES+
7.18e6
100
%
0
245.1284
386.1456
246.1313
267.1100
387.1485
364.1646
100.0773
146.0605
245.0703
268.1123
402.1193
511.2298
630.2662
749.2318
749.3035
750.3067
751.3051
817.2945
907.5753
1048.5771
1112.4548
1191.6982
m/z
O
N
3b

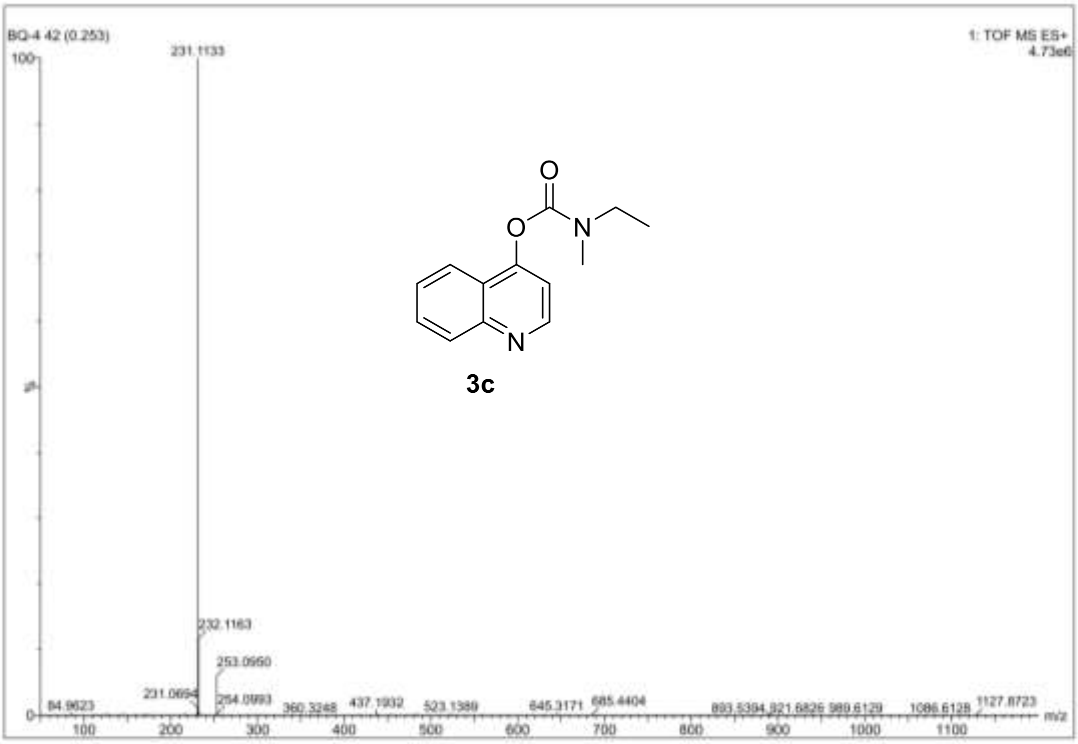
BQ-4 42 (0.253)
1: TOF MS ES+
4.73e6
231.1133
232.1163
253.0950
231.0694
254.0993
84.9623
360.3248
437.1932
523.1389
645.3171
685.4404
893.5394
921.6826
989.6129
1086.6128
1127.8723
m/z
%

3c

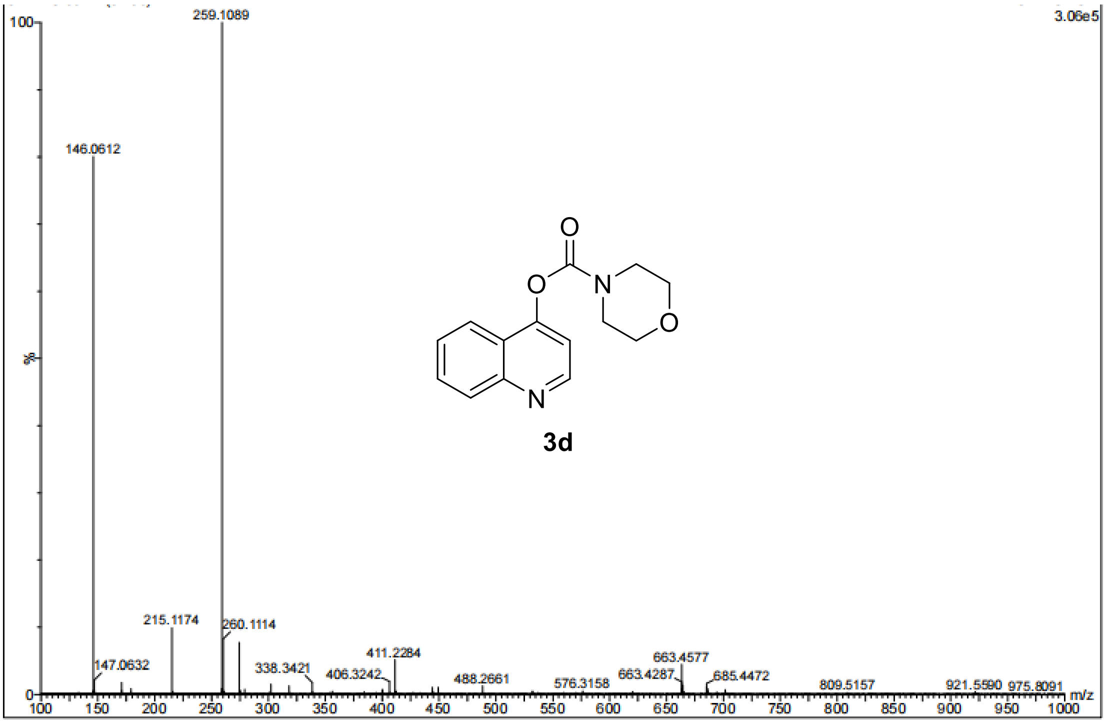
3.06e5
259.1089
146.0612
215.1174
260.1114
147.0632
338.3421
406.3242
411.2284
488.2661
576.3158
663.4287
663.4577
685.4472
809.5157
921.5590
975.8091
m/z
%

3d

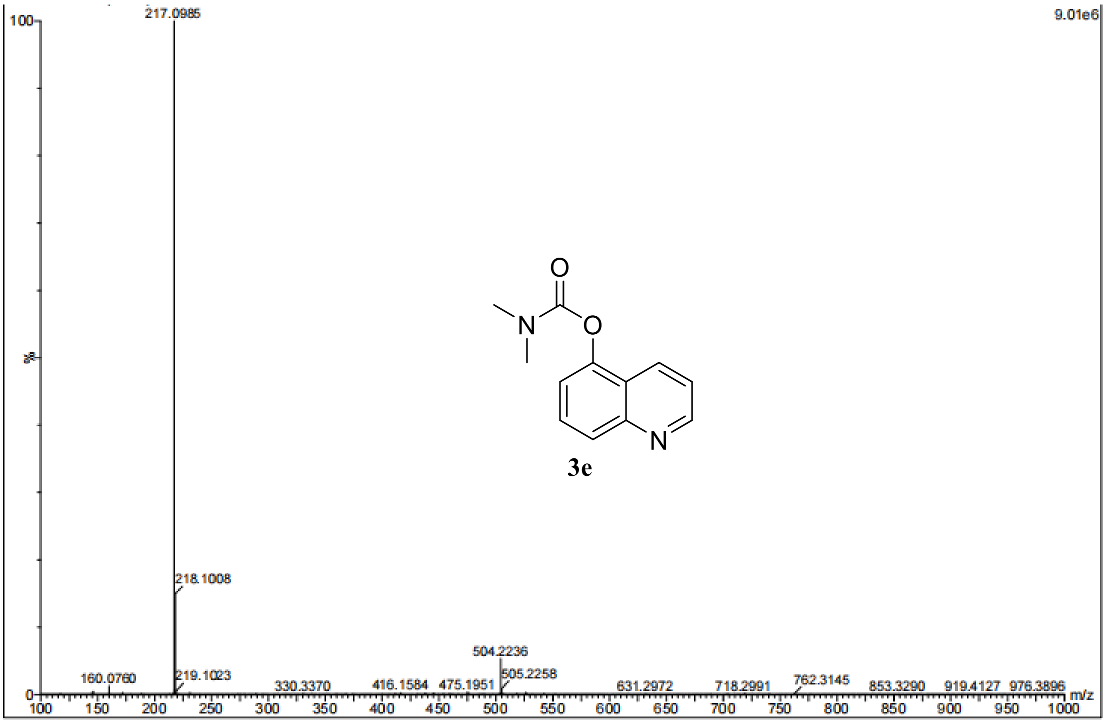
217.0985
9.01e6
100
%
0
218.1008
504.2236
505.2258
160.0760
219.1023
330.3370
416.1584
475.1951
631.2972
718.2991
762.3145
853.3290
919.4127
976.3896
m/z
100
150
200
250
300
350
400
450
500
550
600
650
700
750
800
850
900
950
1000
O
N
O
N
3e

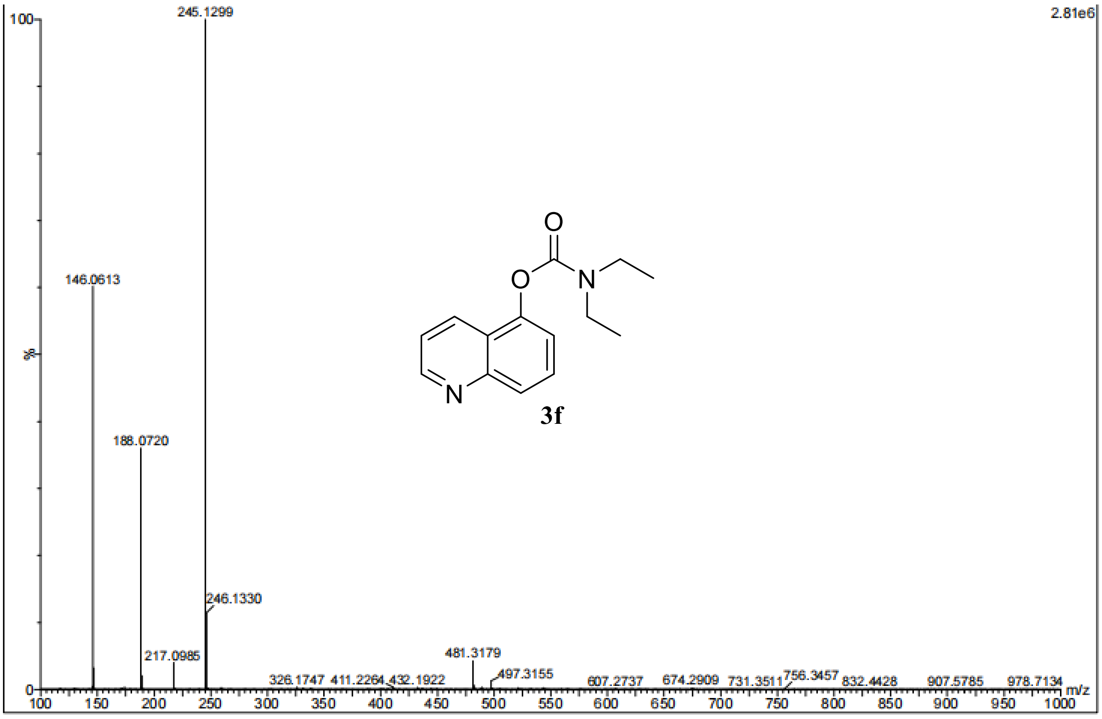
245.1299
2.81e6
100
%
0
146.0613
188.0720
246.1330
217.0985
481.3179
497.3155
326.1747
411.2264
432.1922
607.2737
674.2909
731.3511
756.3457
832.4428
907.5785
978.7134
m/z
100
150
200
250
300
350
400
450
500
550
600
650
700
750
800
850
900
950
1000
O
O
N
N
3f

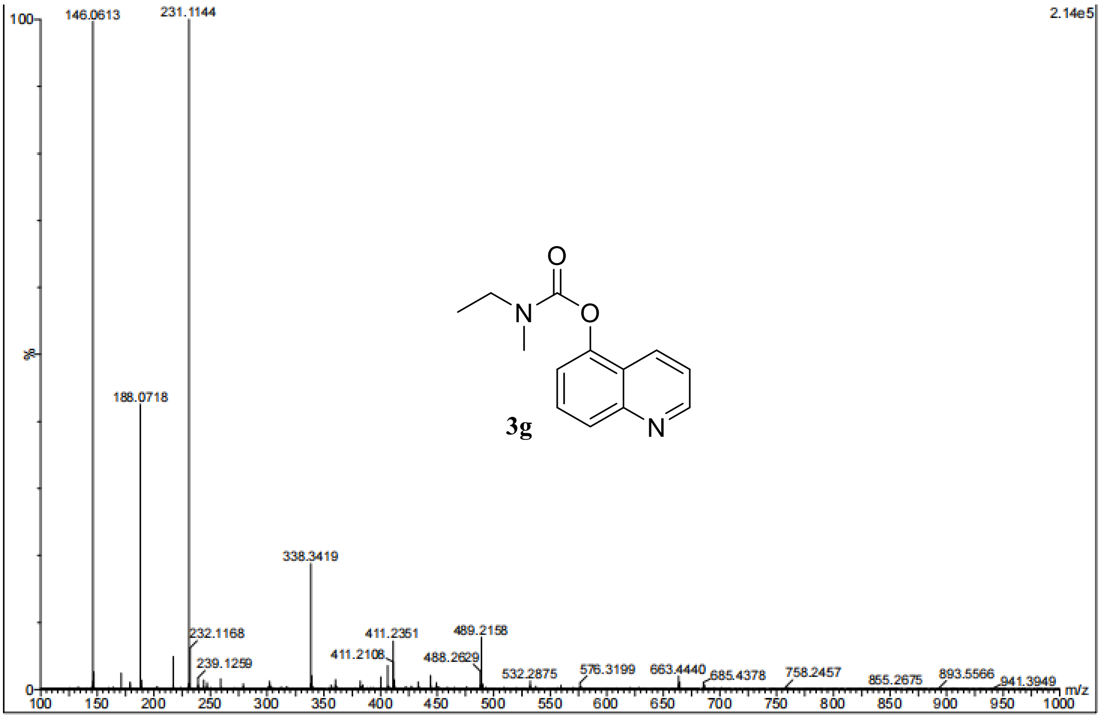
100
146.0613
231.1144
2.14e5
%
188.0718
3g
338.3419
232.1168
411.2351
489.2158
411.2108
488.2629
239.1259
532.2875
576.3199
663.4440
685.4378
758.2457
855.2675
893.5566
941.3949
m/z
0
100 150 200 250 300 350 400 450 500 550 600 650 700 750 800 850 900 950 1000

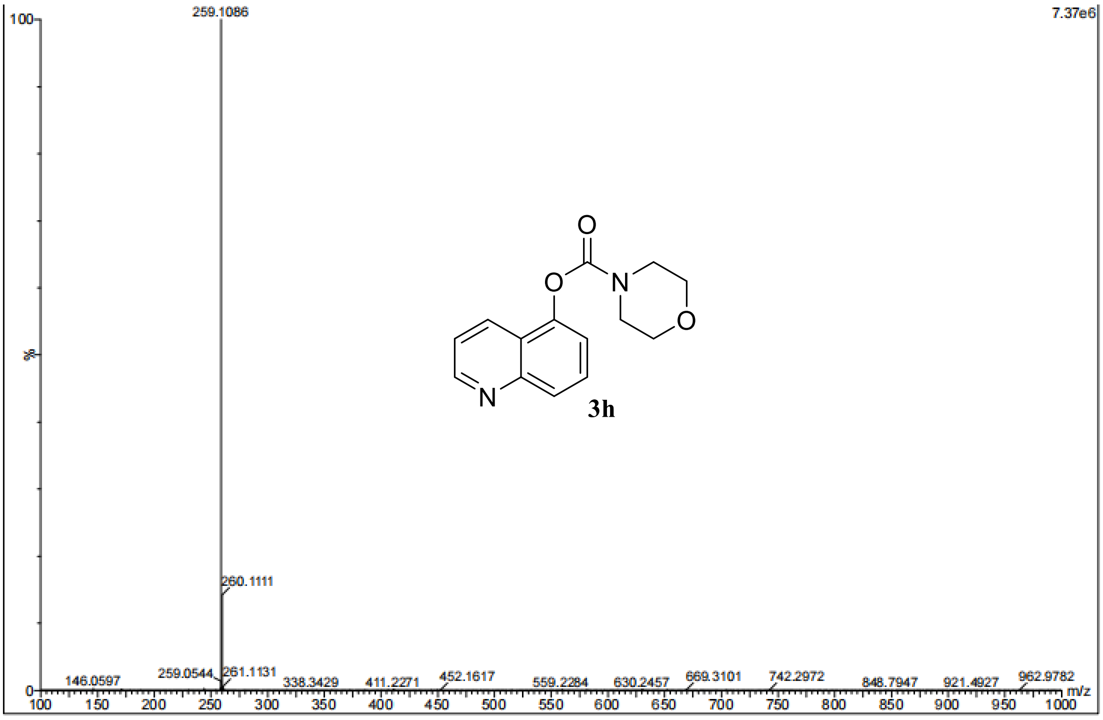
100
259.1086
7.37e6
%
3h
260.1111
146.0597
259.0544
261.1131
338.3429
411.2271
452.1617
559.2284
630.2457
669.3101
742.2972
848.7947
921.4927
962.9782
m/z
0
100 150 200 250 300 350 400 450 500 550 600 650 700 750 800 850 900 950 1000

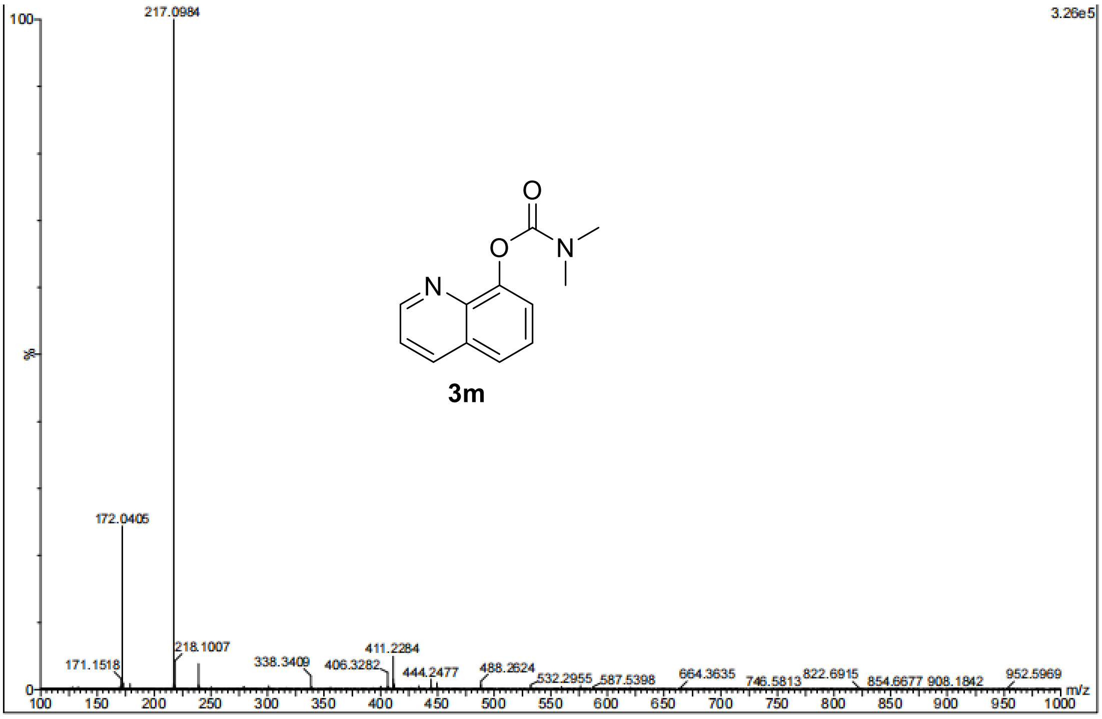
217.0984
3.26e5
100
%
0
172.0405
171.1518
218.1007
338.3409
406.3282
411.2284
444.2477
488.2624
532.2955
587.5398
664.3635
746.5813
822.6915
854.6677
908.1842
952.5969
m/z
100 150 200 250 300 350 400 450 500 550 600 650 700 750 800 850 900 950 1000
O
O
N
N
3m

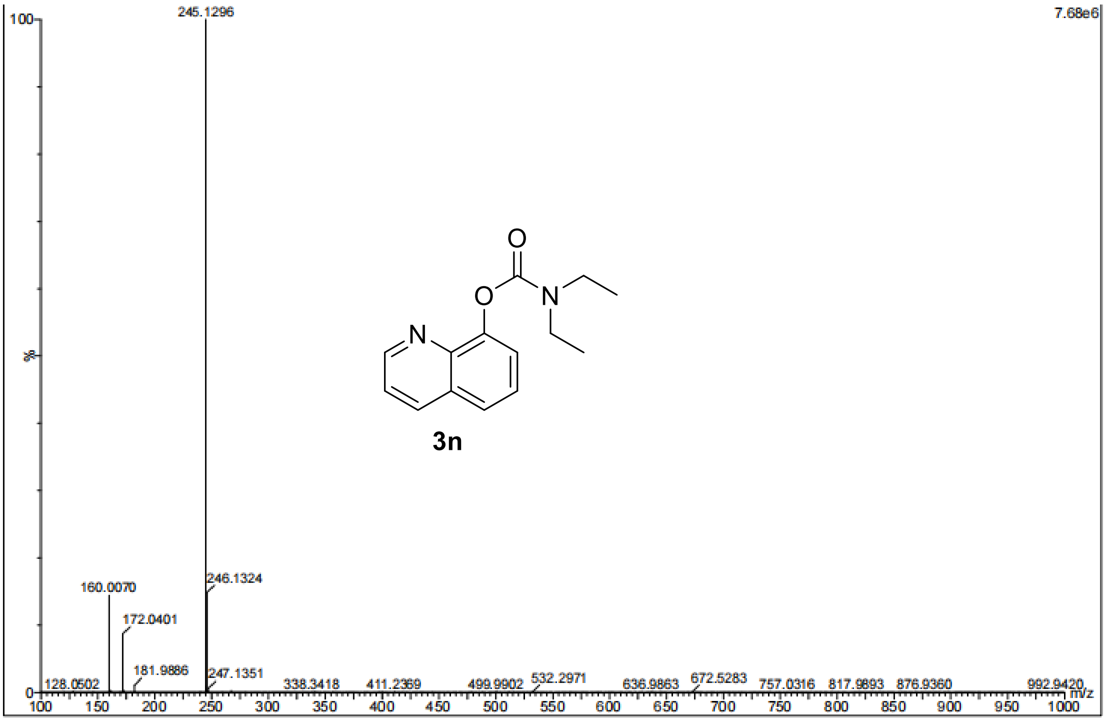
245.1296
7.68e6
100
%
0
160.0070
172.0401
246.1324
128.0502
181.9886
247.1351
338.3418
411.2369
499.9902
532.2971
636.9863
672.5283
757.0316
817.9893
876.9360
992.9420
m/z
100 150 200 250 300 350 400 450 500 550 600 650 700 750 800 850 900 950 1000
O
O
N
N
3n

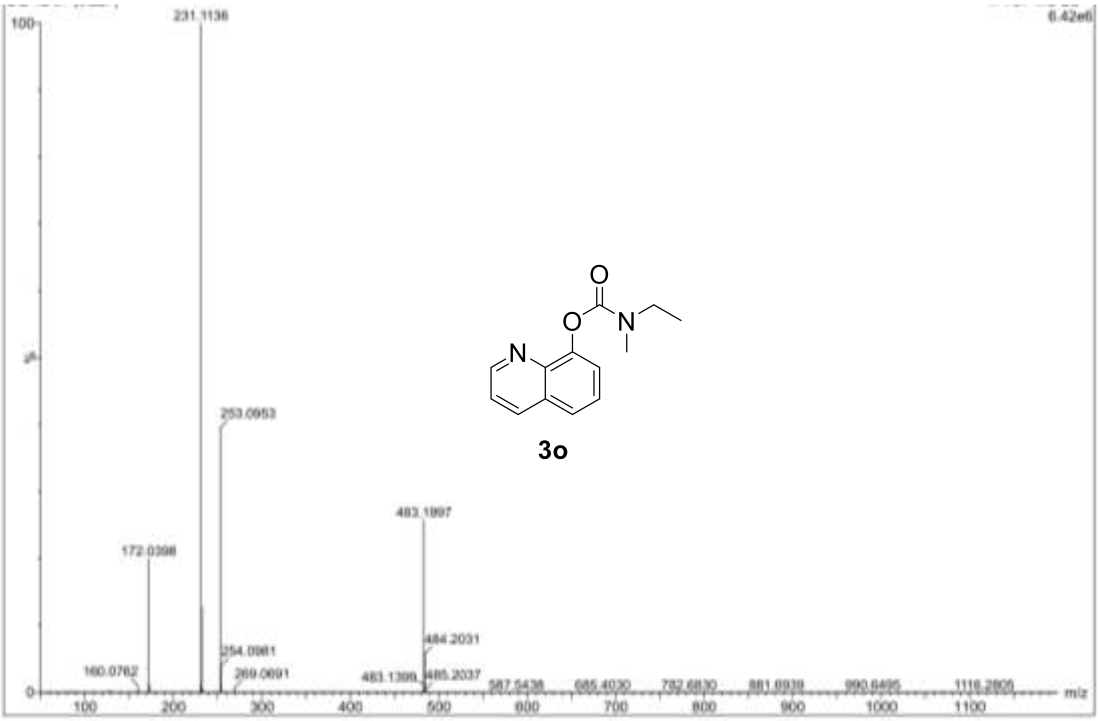

231.1136
6.42e6
100
%
253.0953
483.1997
172.0398
484.2031
254.0981
160.0762
269.0691
483.1399
485.2037
0
100
200
300
400
500
600
700
800
900
1000
1100
m/z
3o

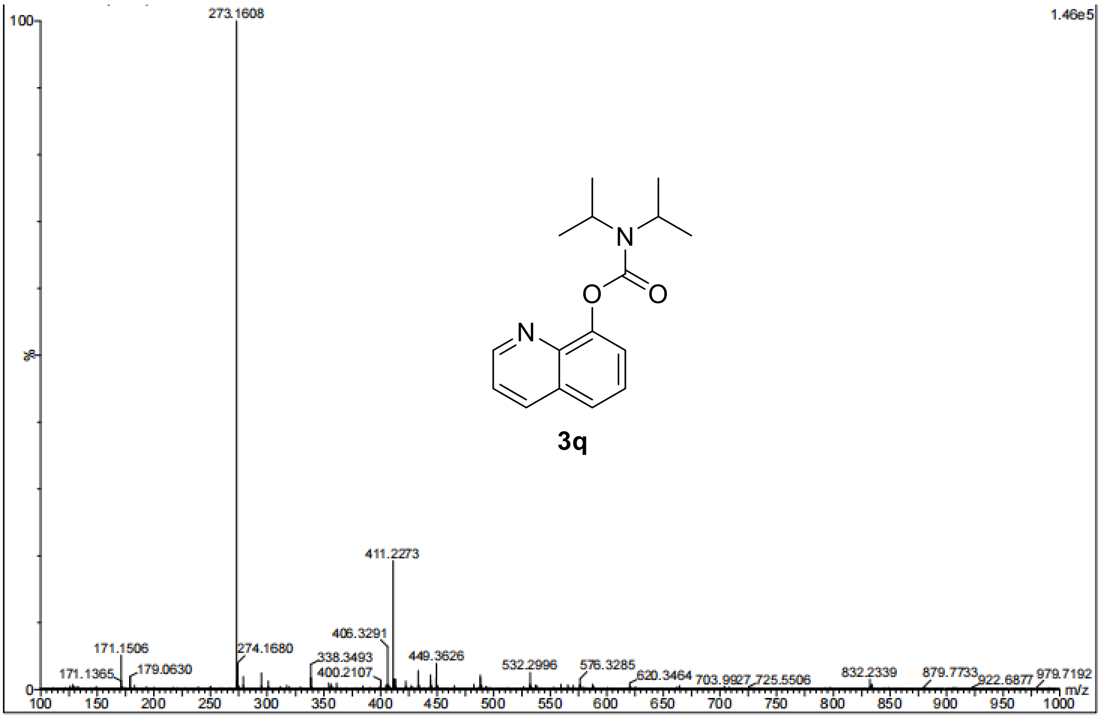

273.1608
1.46e5
100
%
411.2273
171.1506
274.1680
406.3291
449.3626
338.3493
400.2107
171.1365
179.0630
532.2996
576.3285
620.3464
703.9927
725.5506
832.2339
879.7733
922.6877
979.7192
0
100
150
200
250
300
350
400
450
500
550
600
650
700
750
800
850
900
950
1000
m/z
3q
